# Recent Advances in Metal Chalcogenides (MX; *X* = *S*, *Se*) Nanostructures for Electrochemical Supercapacitor Applications: A Brief Review

**DOI:** 10.3390/nano8040256

**Published:** 2018-04-19

**Authors:** Jayaraman Theerthagiri, K. Karuppasamy, Govindarajan Durai, Abu ul Hassan Sarwar Rana, Prabhakarn Arunachalam, Kirubanandam Sangeetha, Parasuraman Kuppusami, Hyun-Seok Kim

**Affiliations:** 1 Centre of Excellence for Energy Research, Sathyabama Institute of Science and Technology, Chennai 600119, India; j.theerthagiri@gmail.com (J.T.); durainayak@gmail.com (G.D.); pkigcar@gmail.com (P.K.); 2 Division of Electronics and Electrical Engineering, Dongguk University-Seoul, Seoul 04620, Korea; karuppasamyiitb@gmail.com (K.K.); a.hassan.rana@gmail.com (A.u.H.S.R.); 3 Electrochemistry Research Group, Chemistry Department, College of Science, King Saud University, Riyadh 11451, Saudi Arabia; prabhunittmsc@gmail.com; 4 Biomaterial Research Lab, DKM College for Women, Vellore 632001, India; shopna.san@gmail.com

**Keywords:** capacitance, electrode materials, selenides, supercapacitor, sulfides

## Abstract

Supercapacitors (SCs) have received a great deal of attention and play an important role for future self-powered devices, mainly owing to their higher power density. Among all types of electrical energy storage devices, electrochemical supercapacitors are considered to be the most promising because of their superior performance characteristics, including short charging time, high power density, safety, easy fabrication procedures, and long operational life. An SC consists of two foremost components, namely electrode materials, and electrolyte. The selection of appropriate electrode materials with rational nanostructured designs has resulted in improved electrochemical properties for high performance and has reduced the cost of SCs. In this review, we mainly spotlight the non-metallic oxide, especially metal chalcogenides (MX; *X* = *S*, *Se*) based nanostructured electrode materials for electrochemical SCs. Different non-metallic oxide materials are highlighted in various categories, such as transition metal sulfides and selenides materials. Finally, the designing strategy and future improvements on metal chalcogenide materials for the application of electrochemical SCs are also discussed.

## 1. Introduction

A substantial global upsurge in the depletion of fossil fuels from the rapid growth of global economy has generated two vital concerns: the first is the exhaustion of existing fossil fuel reserves, and the second is associated with an increase in greenhouse gas emissions, in particular, and environmental pollution, in general. Hence, it is necessary to develop and commercialize sustainable environment friendly energy sources and their related technologies are being developed globally as a matter of urgency [1,2,3,4,5,6]. Also, the development of associated energy conversion devices to gather these intermittent energy sources efficiently is in demand. In this specific backdrop, electrochemical supercapacitors (SCs) have overriding importance because of their exceptional power density and storage properties compared to other contemporary energy storage devices. SCs have a number of great advantages including long life cycle, high power density, high efficiency, high specific capacitance, flexible operating temperature, and environmental friendliness. Moreover, they are quickly charged with fast power delivery and are capable to bridge the gap between batteries and conventional capacitors [7,8,9,10,11,12]. 

SCs are used in applications which require many charge and discharge cycles, rather than long-term compact energy storage within hybrid vehicles and electronic systems. Depending on the mode of energy storage in SCs, they are classified into three types, namely electrical double layer capacitors (EDLCs), pseudocapacitors, and hybrid capacitors. EDLCs are based on the working principle of the charge being stored electrostatically within the electric double layer formed at the interface of two electrodes. Generally, EDLCs use carbon-based materials, such as activated nanoporous carbon, carbon aerogel, carbon nanosheets, carbon nanotubes (CNTs), and graphene, to store energy [13].

Pseudocapacitors are another type of SC in which electrical energy storage is based on the working principle of faradaic charge transfer between the electrode and the electrolyte by reduction and oxidation reactions. Metal oxides (IrO_2_, RuO_2_, NiO, MnO_2_, MoO, V_2_O_5_, Fe_3_O_4_, etc.), metal chalcogenides (MS_2_, MSe_2_), metal nitrides (VN, RuN, MoN, TiN, etc.), and conducting polymers (polyaniline, polythiophene, polypyrrole (PPy), etc.) are the electrode materials which have been employed in pseudocapacitors [14,15]. Hybrid-type SCs are a combination of both EDLCs and pseudocapacitors. The best electrochemical properties for high-performance SCs can be grabbed by opting reasonable electrode materials with aptly chosen electrolytes and nanostructured designs. An ideal electrolyte should consist of high ionic conductivity and thermal stability, high chemical and electrochemical stability; chemical and electrochemical inertness to SC components, such as electrodes, current collectors, and packages. In real-world terms, it is exceptionally difficult for any electrolyte to meet all the above requirements, and each electrolyte has its own advantages and disadvantages [16]. The electrolytes for SC strongly depends on the nature, including (a) the ion type and size; (b) the ion concentration and solvent; (c) the interaction between the ion and the solvent; (d) the interaction between the electrolyte and the electrode materials; and (e) the potential window, all have an influence on the double layer capacitance and pseudocapacitance. Furthermore, the interactions between the ion and the solvent and between the electrolyte and the electrode material can affect the lifetime and self-discharge of SCs [17]. Hence, electrolytes are identified as one of the most persuasive components in the performance of SCs. However, the nanostructure designs have the ability to improve electrochemical reaction efficiency and utilization of active materials with improved energy and power densities. This is for the reason that, despite tremendous improvements in the material science of the electrodes, not many studies have reported metal chalcogenide-based nanostructured electrode materials for electrochemical SCs.

The electrochemical performance of an SC is estimated by the specific capacitance, energy density, and power density, which are evaluated according to the Equations (1)–(4) [18,19,20].

The electrode materials’ specific capacitance (in F·g^−1^) is calculated via a current-voltage (CV) analysis:

(1) $$C=\frac{\int Idv}{2mv\Delta V}$$

where *m* is mass of the used electrode material (g), *I* is the voltammetric current, ∆*V* is the potential window (V), and *v* is the scan rate (mV·s^−1^), respectively. The electrode materials’ specific capacitances are evaluated from a (CD) analysis:

(2) $$C=\frac{I\Delta t}{m\Delta V}$$

where *I* is the discharging current (A), *t* is the time (s), and *V* is the potential difference (V), respectively. Furthermore, the energy and power densities are calculated by

(3) $$E=\frac{I\Delta V\Delta t}{m}$$

and

(4) $$P=\frac{I\Delta V}{m}$$

respectively. In the aforementioned Equations (3) and (4), the *I*, ∆*V*, ∆*t*, and *m* are the current potential difference, discharging time, and mass of an electroactive material, respectively.

Criteria for electrode materials selection are

(i) Multiple oxidation states
(ii) Superior electrical conductivity
(iii) High surface area & chemical stability
(iv) Electrochemical activity (electrolyte ions can freely interact into the electrode surface)

To improve the capacitance of supercapacitors, four key factors are required:

(i) Doping of the metals to increase the conductivity and redox activity
(ii) A wide potential window
(iii) High surface area for the redox reaction
(iv) High charge/discharge rate

In the present review, the recent advances in the fabrication of metal sulfides and metal selenide-based nanostructured electrode materials for electrochemical SCs are discussed. Finally, the benefits of both metal sulfide- and selenide-based nanostructured electrode materials in the designing strategy for electrochemical SC applications are also systematically presented.

## 2. Metal Chalcogenides for Electrochemical SCs

The industrially vital and scientifically significant metal chalcogenides (MCs) (S, Se, and Te) have received a great deal of attention in the past two decades due to their anisotropic property. In general, transition elements of groups IV to VII B combine with VI A group elements, such as S, Se, and Te to form binary stable layered crystalline structures [21]. These layered transition MCs possess the general formula of MX_2_, where M is a transition element in groups IV B (Ti, Zr, Hf), V B (V, Nb, Ta), VI B (Mo, W), or VII B (Tc, Re) and X is a chalcogen atom in the VI A group (S, Se, Te). The structure and properties of most of the transition MCs almost resemble semimetal pristine graphene, except for the band gap [22], which is nearly zero in pristine graphene whereas in transition MCs, it depends on the elemental combination, the number of layers, and the presence or lack of adopting atoms. Hence, their band gap values lie between 0 and 2 eV. Due to the variation in band gap, different transition MC structures are tunable, and so have become industrially important materials [23]. 

In this part of the review article, we particularly describe the application of nanostructured transition MCs in electrochemical SCs. They have gained considerable attention due to their high specific power, and long stability and life cycle, and they offer better safety tolerance relative to batteries in a wide range of applications in consumer electronics, electric tools, buffer powers, hybrid electronic vehicles, and so forth [22]. On the other hand, MCs have been applied in the fields of fuel cells, solar cells, light-emitting diodes, sensors, lithium-ion batteries, electrocatalysts, thermoelectric devices, and memory devices, as well as being widely utilized in SCs, due to their excellent properties. These include (i) improved life cycle; (ii) flexibility; (iii) providing additional reactive sites and catalytic activity; (iv) improving conductivity as well as reduction of inner resistance and ohmic loss; (v) short path lengths for electron transport; and (vi) displaying quantum-sized effects. Furthermore, we describe the future promising areas of transition metal group sulfides and selenide nanostructures covering both their properties and their applications in SCs. Specifically, metal sulfides exhibit greatly improved electrochemical performance, which largely originates from their higher electronic conductivity, higher electrochemical activity, and mechanical and thermal stability. On the other hand, it has been well reported that the performance of electrochemical energy storage devices depends greatly on the crystalline phase, size of the electroactive materials, structural and morphological features, and composition and the design of electrodes [22]. Metal selenides, as a new class of battery-like electrode materials, have gained increasing interests as promising supercapacitor electrode materials, not only possessing rich redox chemistry, but also better electronic conductivity, and mechanical and thermal stability. Compared to metal sulfides, metal selenides are for less reported than that of metal sulfides. The details are presented herein.

## 3. Transition Metal Sulfides

### 3.1. Nickel Sulfides

In recent years, nanometer-sized metal sulfides have played a significant role in the field of electronics, especially optical and optoelectronic devices, due to their distinct excellent physical and chemical properties. Certainly, nickel sulfide is of particular interest because of its different phases, such as NiS, Ni_3_S_2_, NiS_2_, Ni_3_S_4_, Ni_7_S_10_, and Ni_9_S_8_, and its different morphologies [24]. However, the different phases and morphologies of nickel sulfides sometimes coexist as a combination of more than two different phases [25]. Hence, obtaining an even morphology with pure nickel sulfides is still a challenge that has attracted a great deal of attention. Some of the important phases of nickel sulfides and their application in SCs are briefly discussed under the following subsections. 

(a) Ni_3_S_2_

In the midst of the different types of nickel sulfides, Ni_3_S_2_ has exhibited a better performance as an electrode material for energy storage devices, due to its different types of morphology and advantages, including its low capital cost, high specific capacitance, and simple synthesis route. These are anticipated to help it meet the increasing necessities of energy storage systems, especially for SCs [25]. In addition, it occurs abundantly in nature as minerals in the form of heazlewoodite. Hence, in recent years, it has been investigated widely for SC applications. However, Ni_3_S_2_, unfortunately, has low conductivity, which restricts the fast electron transport required for high rate capability, and can even act as an insulator. This sort of issue has been overcome by way of incorporating highly conductive electrode materials in the pseudocapacitive Ni_3_S_2_ material.

**Figure 1:**
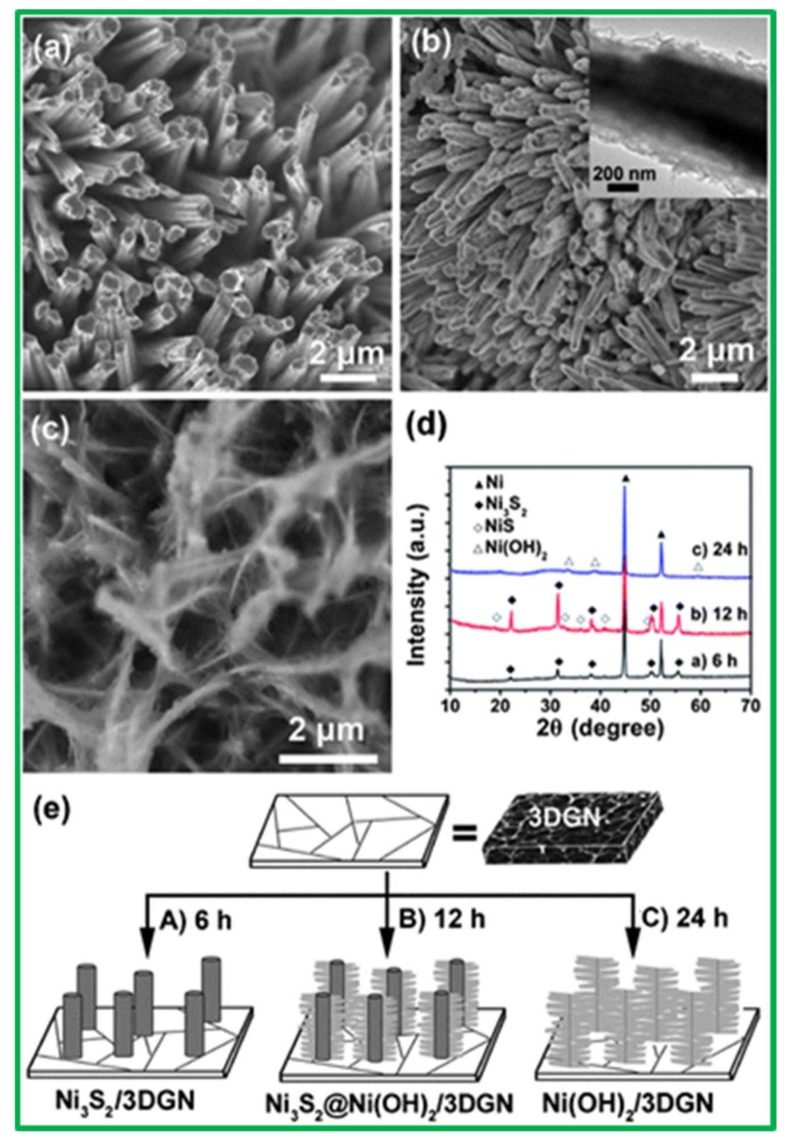
Scanning electron microscopy images of Ni_3_S_2_ nanorods obtained at different hydrothermal reaction times: (**a**) 6 h; (**b**) 12 h; and (**c**) 24 h (the inset in (**b**) is a magnified image of the Ni_3_S_2_@Ni(OH)_2_/3-D-GN structure); (**d**) X-ray diffraction (XRD) patterns of the samples shown in (**a**–**c**); and (**e**) a proposed mechanism for the growth of the Ni_3_S_2_@Ni(OH)_2_/3-D-GN structure. Reproduced with permission from [31]. Royal Society of Chemistry, 2016.

Chou et al. [26] first synthesized the flaky Ni_3_S_2_ nanostructure on Ni-foam by a simple potentiodynamic deposition method and employed it for SCs. This material showed a maximum specific capacitance of 717 F·g^−1^ at 2 A·g^−1^ rate in 1 M KOH solution with remarkable capacitance retention of 91%. On the other hand, Karthikeyan et al. [27] used a one-pot hydrothermal synthesis method of Ni_3_S_2_ to increase the electrochemical properties and specific capacitance of Ni_3_S_2_ further. They grew hierarchical Ni_3_S_2_ nanostructures in a Ni foam cell and evaluated its capacitance behavior. The cell offered a maximum specific capacitance of 1293 F·g^−1^ at a current density of 5 mA·cm^−2^. Moreover, a different kind of preparation method has been extensively studied and reported for other similar type of electrode materials [28,29]. Zhou et al. [30] further used a hydrothermal method to synthesize Ni(OH)_2_ nanosheets coated onto single-crystal Ni_3_S_2_ nanorods grown on the surface of three-dimensional (3-D) graphene nanosheets (Ni_3_S_2_@Ni(OH)_2_/3-D-GN), which were able to achieve a relatively high capacitance of 1277 F·g^−1^ at 2 mV·s^−1^ and 1037.5 F·g^−1^ at 5.1 A·g^−1^. They also investigated the structural evaluation of Ni_3_S_2_@Ni(OH)_2_/3-D-GN with respect to hydrothermal reaction time, and concluded that as the reaction time increases from 6 h to 12 h, the evolution of the structure from Ni_3_S_2_ nanorods to Ni_3_S_2_@Ni(OH)_2_ occurred, followed by conversion to pure Ni(OH)_2_ nanosheets. After a hydrothermal reaction time of 6 h, Ni_3_S_2_ nanorods were obtained, as exhibited in Figure 1.

Later on, Zhu et al. [32] reported the preparation of Ni_3_S_2_ nanosheets on a CNT backbone with a specific capacitance of 514 F·g^−1^ at a current density of 4 A·g^−1^ and excellent cycling stability. Likewise, Pan et al. [33] designed and compared the capacitance behavior between Ni_3_S_2_ and Ni_3_S_2_/graphene on Ni-foam. Obviously, compared to pristine Ni_3_S_2_, the Ni_3_S_2_/graphene nanocomposites showed better electrochemical behavior and achieved a specific capacitance value of around 278.3 F·g^−1^ for the first 20 cycles. Afterwards, the capacitance started to decrease to 230.6 F·g^−1^ over 35 cycles, and finally reached 223 F·g^−1^ until 50 cycles, which might have been due to the detachment of electrode material from the Ni-foam.

To improve the specific capacitance of Ni_3_S_2_/graphene composites, a simple process controlled by adjusting the extent of sulfidation was proposed by Ou et al. [34] who achieved the highest specific capacitance of 1022 F·g^−1^. The same group also studied the one-step hydrogen reduction synthesis of Ni_3_S_2_/graphene composites reported elsewhere [35]. Moreover, the biomolecule-assisted hydrothermal synthesis of Ni_3_S_2_ nanospheres/reduced graphene oxide (Ni_3_S_2_/rGO) nanocomposites was investigated using l-cysteine as the reducing agent, and their application to SCs characterized [36]. They displayed very high specific capacitances of 1169 F·g^−1^ and 761 F·g^−1^ at 5 A·g^−1^ and 50 A·g^−1^ current rates, respectively, with good cycling stability, while bare Ni_3_S_2_/rGO on Ni-foam offered a specific capacitance of 2188.8 F·g^−1^ at 2.9 A·g^−1^ [37].

In recent times, a series of Ni_3_S_2_ nanowires, such as Ni_3_S_2_-Ni, Ni_3_S_2_-NiS, and Ni_3_S_2_-NiS-Ni, have been grown on nickel nanowire templates, and their capacitance behavior compared elaborately [38]. Among these, Ni_3_S_2_-NiS nanowires presented superior redox reactivity with a high specific capacitance of 1077.3 F·g^−1^ at 5 A·g^−1^, due to their excellent aspect ratio and electrical conductivity. On the contrary, the other two nanowire electrodes (Ni_3_S_2_-Ni and Ni_3_S_2_-NiS-Ni) possessed 100% capacitance retention compared to the Ni_3_S_2_-NiS electrode (76.3%). A rationally designed two-step method to fabricate self-supported Ni_3_S_2_ nanosheet arrays on a metal-organic framework has been investigated by Chen et al. [39] who achieved a maximum specific capacitance of 200 F·g^−1^ at a current density of 10 A·g^−1^.

(b) NiS

As discussed earlier, uniform morphology with a pure phase of nickel sulfide is still a challenge, and currently, plenty of research is focused on resolving this problem [40]. Nevertheless, few studies have dealt with morphological control during the synthesis of the NiS and NiS_2_ phases with a pyrite structure [41,42]. In addition, those consisting of nickel sulfide phases are less toxic and highly abundant in nature, and possess high redox activity [43,44,45,46]. For instance, flower-like β-NiS was successfully synthesized and reported by Yang et al. [47], in which the electrodes displayed a specific capacitance of 966 F·g^−1^ at a current rate of 0.5 A·g^−1^. Similarly, Wang et al. [48] prepared one-dimensional (1-D) (110)-oriented NiS nanorods with a high specific capacitance of 1403.8 F·g^−1^ at a current density of 1 A·g^−1^. This high specific capacitance of the electrode material might have been due to the designed 1-D electron-transport pathway and large specific surface area of NiS. Likewise, successful SC performances of α-NiS and β-NiS were reported by Wei et al. [49].

However, the pure phases of these electrodes suffer from poor cycling stability owing to the agglomeration and pulverization of NiS during consecutive cycling of the CD process. The cycling stability of NiS electrodes has been improved by changing the experimental conditions, and including conducting nanomaterials along with a NiS matrix, as reported earlier, some of which are listed later. The phase-controlled synthesis of α-NiS embedded in carbon nanorods was synthesized by Sun et al. [50]; the electrodes delivered a high electrochemical stability with 100% capacitance retention with a specific capacitance of 1092 F·g^−1^ at 1 A·g^−1^. Similarly, NiS nanoparticles on Ni-foam, [51,52,53] activated carbon, [53] N-doped carbon fiber aerogels [46], and rGO [54], have been reported recently.

(c) Ni_3_S_4_

**Figure 2:**
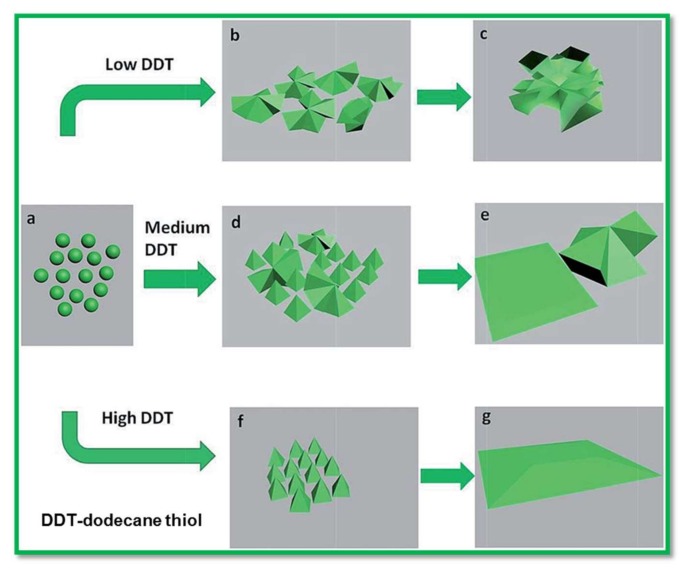
Schematic illustration for the formation of 3-D Ni_3_S_4_ nanosheet frames and Ni_3_S_4_ sheets. Reproduced with permission from [55]. Royal Society of Chemistry, 2015.

One of the rarely reported nickel sulfide phases, Ni_3_S_4_, exists in nature as polydymite. Still, the scientific community is facing the challenge to obtain the purest phase of Ni_3_S_4_ by conventional solid-state reactions for SC applications. Hence, to date, the electrochemical properties of Ni_3_S_4_ remain hidden. Only a few studies in the literature discussed earlier are on Ni_3_S_4_ for SC applications. In recent times, Zhang et al. [55] prepared the 3-D rigid Ni_3_S_4_ nanosheet frames by controlled solvothermal synthesis, and evaluated their electrochemical performances for SC applications. Interestingly, the 3-D rigid Ni_3_S_4_ nanosheet frames possessed better capacitance performances than that of flat Ni_3_S_4_. The 3-D rigid Ni_3_S_4_ nanosheet frames achieved a maximum capacitance value of 1213 F·g^−1^, which was due to high free volume and high compressive length. The proposed mechanism for both flat Ni_3_S_4_ and 3-D Ni_3_S_4_ nanosheet frames is schematically represented in Figure 2. Furthermore, the synergistic effects of the layered Ni_3_S_4_, MoS_2_, and conductive carbon fibers were analyzed by Huang et al. [56] who reported a capacitance value of 1296 with 96.2% capacitance retention. Similarly, the Ni_3_S_4_@amorphous MoS_2_ nanosphere electrodes have exhibited a high specific capacitance of 1440.9 F·g^−1^ at 2 A·g^−1^ [57].

Easily self-assembled Ni_3_S_4_-MoS_2_ hetero-junction electrode materials assisted by an ionic liquid 1-butyl-3-methylimidazolium thiocyanate have been prepared for the first time with the electrode attaining high specific capacitance of 985.21 F·g^−1^ at a current density of 1 A·g^−1^ [58]. The role of ionic liquid in this hetero-junction electrode synthesis was that it provides a sulfur source for the sulfidation reaction, and also influences the formation of Ni_3_S_4_-MoS_2_ with different precursor reactions. Other phases of nickel sulfides, such as Ni_9_S_8_ [59] and NiS_2_ [24], were also produced but rarely reported for SC applications, due to their unstable phase nature.

### 3.2. Copper Sulfide

The inexpensive, naturally abundant functional semiconductor copper sulfide is available as different phases, such as chalcocite (Cu_2_S), villamaninite (CuS_2_), djurleite (Cu_1.95_S), anilite (Cu_1.75_S), and covellite (CuS) in nature [60,61]. Among these, CuS has been the extensively studied, and is used in energy storage and conversion devices, gas sensors, and photocatalysts [62]. Furthermore, different approaches have been adopted to synthesize CuS, including solvothermal synthesis, microemulsions, and surfactant templating, due to its low capital cost [63,64]. 

In this section, we briefly discuss the salient features and potential applications of CuS in the field of electrochemical SCs. Studies on the electrochemical behavior of CuS are very limited, and so an investigation into its use as an electrode material is highly significant. Recently, it has been reported as a suitable SC electrode material, due to its high theoretical capacitance [64,65,66,67]. For example, Peng et al. [68] synthesized CuS with different morphologies using a low-temperature solvothermal method, and employed it for SC applications. The high surface area flower-like CuS provided a good specific capacitance of 597 F·g^−1^ with an excellent discharging rate and cycling stability. The sonochemical-assisted synthesis of CuS has been studied elaborately, and yielded a specific capacitance of 62.77 F·g^−1^ at 5 mV·s^−1^ [69].

The important metal chalcogenide CuS provides an electronic conductivity of 10^−3^ S·cm^−1^ and theoretical specific capacity of 561 mA·h·g^−1^. However, this is not favorable for SC applications because pure CuS is a semiconductor with relatively low conductivity when compared to carbon nanomaterials and conducting polymers, and its volume change during cycling causes poor cycling stability [70]. Hence, it is desirable to geometrically control the preparation of CuS composites and combine them with electronically conductive substance to enhance SC performance greatly.

**Figure 3:**
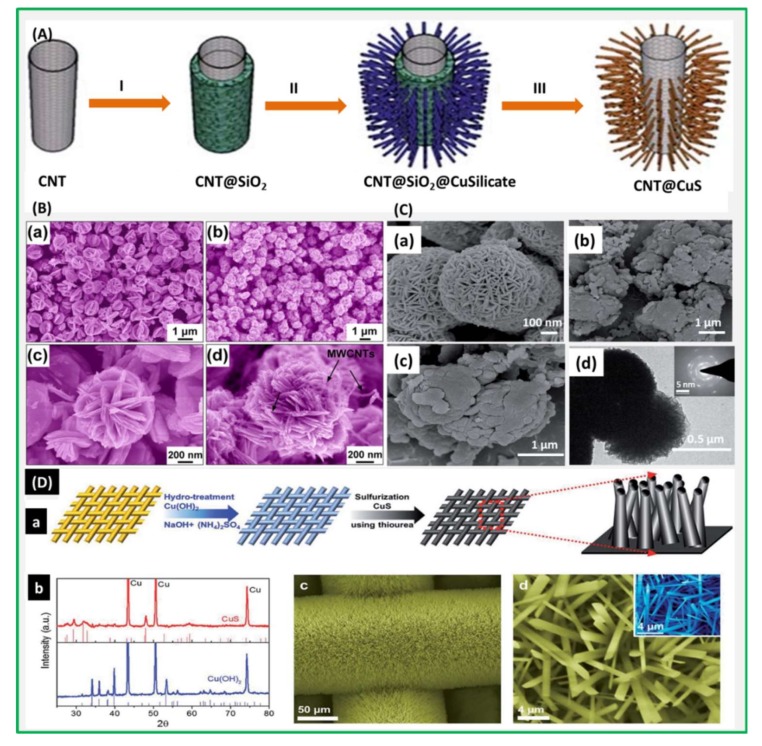
(**A**) Schematic illustration of the formation of carbon nanotube (CNT)@CuS by a template-engaged conversion route: (I) Uniform coating of a silica layer on CNT; (II) growth of copper silicate nanoneedles on the silica layer; and (III) chemical conversion to CNT@CuS with the silica layer simultaneously eliminated. Reproduced with permission from [62]. Royal Society of Chemistry, 2012; (**B**) SEM images of CuS (**a**,**c**); CuS/CNT composites (**b**,**d**). Reproduced with permission from [72]. Springer Nature Publishing Group, 2015; (**C**) FE-SEM images of CuS (**a**) and CuS@PPy composite (CuS content is 16.7 wt %) in low and high magnification (**b**,**c**); TEM images of CuS (**d**) and CuS@PPy composite (CuS content is 16.7 wt %) (**e**). Reproduced with permission from [73]. Royal Society of Chemistry, 2014; (**D**) **(a)** Schematic representation of Synthesis process of CuS NWs; (**b**) XRD patterns of the as-prepared Cu(OH)_2_ and CuS NWs; (**c**) A FE-SEM image of CuS NWs; (**d**) A high-magnification SEM image of CuS NWs. The inset indicates the high-magnification SEM image of Cu(OH)_2_ NWs. Reproduced with permission from [74]. Royal Society of Chemistry, 2016.

Ultrafine CuS nanoneedle arrays grown on a CNT backbone have also been investigated as electrodes for SC applications in the past. Interestingly, these reported 1-D hierarchical electrodes offered better capacitance values with excellent cyclability, owing to the abundant surface area between the electrode and electrolyte. A schematic illustration of the formation of CuS nanoneedles on a CNT backbone is depicted in Figure 3. Later, Huang et al. [71] have applied a different hydrothermal approach to synthesis CuS/MWCNT (multi-walled CNT) electrodes and analyzed its electrochemical performance (2831 F·g^−1^). The CNT-incorporated porous 3-dimensional CuS microsphere composite electrodes had peony-like microspheres with a diameter of 1 μm, and each microsphere was composed of a few tens of bundled nanosheets of 15–30 nm thickness [72]. They showed excellent cyclability and rate capability, with an average reversible capacitance of 1960 F·g^−1^ at 10 mA·cm^−2^. The electrochemical SC performances of different important metal sulfides are tabulated in Table 1.

**Figure 4:**
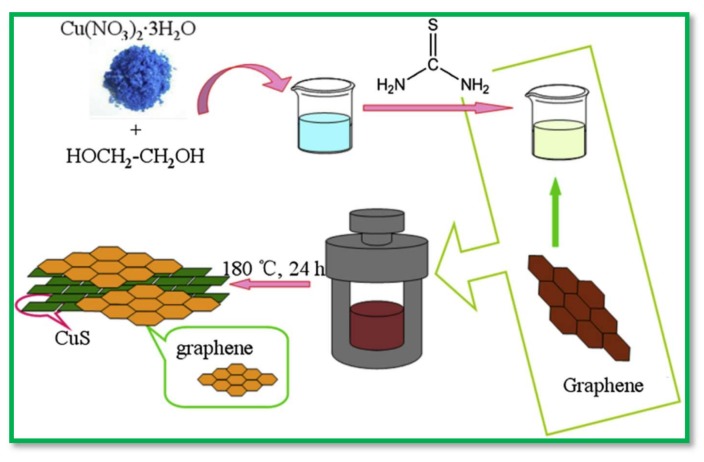
A schematic demonstration for the synthesis of CuS-GO composites. Reproduced with permission from [70]. Elsevier, 2015.

A high-performance SC based on CuS@PPy composite has been developed by in situ oxidation polymerization recently [73]. The composite had uniform spheres with an average thickness of 1 μm, which in turn were composed of plenty of intertwined sheet-like subunits. The electrodes exhibited a high specific capacitance of 427 F·g^−1^ at 1 A·g^−1^. Currently, CuS nanowires on a copper mesh have also served as working electrode in SCs. These CuS-nanowire-based electrodes were free from the binder and conductive material, and had well-arrayed structures with nanosized grains and a high aspect ratio and density. In addition, the other electronically conducting substances like rGO, acetylene black, polyaniline (PANI), and CNTs have also been combined with CuS with the resultant electrodes showing very good capacitance performance and great retention [70,74,75,76,77].For instance, the schematic illustration of synthesis of CuS@rGO composites was displayed in Figure 4 [70]. 

### 3.3. Cobalt Sulfides

In the past decade, cobalt sulfide has received a great deal of interest, due to its applications in versatile fields such as SCs, lithium ion batteries, alkaline rechargeable batteries, magnetic materials, and catalysts [88,89,90,91]. To date, various nanostructures of cobalt sulfide have been examined and reported as electrode materials for SCs. However, the controlled synthesis of cobalt sulfides with high purity and well-defined complex morphology is highly complicated. This may be due to the following factors. (i) Since it exists in nature as different chemical compositions (Co_1−x_S, CoS, CoS_2_, Co_9_S_8_, and Co_3_S_4_), it can easily transform from one phase to another phase; (ii) During preparation, it is very difficult to remove impurities such as cobalt oxide and cobalt hydroxide, because cobalt ions have a very strong affinity to oxygen; (iii) Controlling the reaction temperature is challenging for the reason that cobalt sulfides possess a complicated phase diagram. In order to deal with these factors as well to prepare high purity cobalt sulfide nanostructures, various types of synthetic routes have been employed in the past. There are several reports on the synthesis and electrochemical evaluation of nanostructured cobalt sulfides pertinent to SCs and will be discussed in this section.

(a) Co_3_S_4_

**Figure 5:**
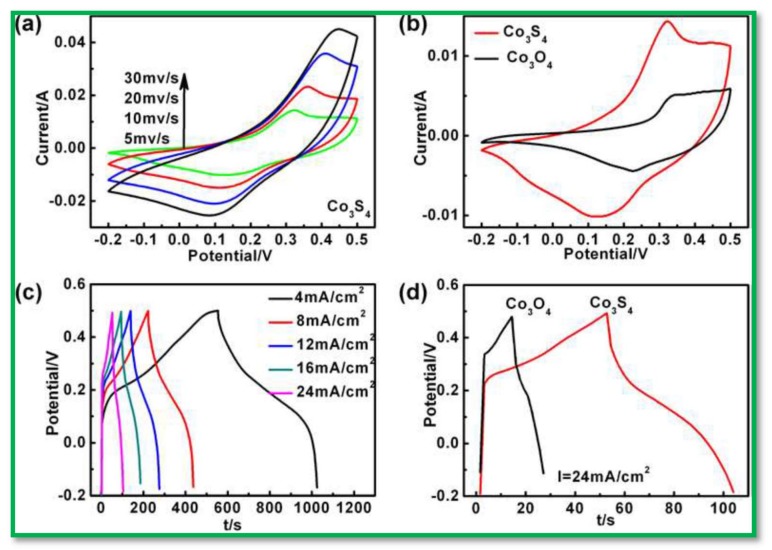
(**a**) The current-voltage (CV) curves of Co_3_S_4_ nanosheet arrays on Ni-foam at different scan rates of 5, 10, 20, and 30 mV·s^−1^; (**b**) CV comparison of the Co_3_S_4_ and Co_3_O_4_ on Ni-foam at the same scan rate of 5 mV·s^−1^; (**c**) The charge-discharge behavior of the Co_3_S_4_ nanosheet arrays at different current densities; (**d**) Comparison of the Co_3_S_4_ nanosheet and Co_3_O_4_ nanowire arrays on Ni-foam with the same areal charge-discharge current of 24 mA·cm^−2^ Reproduced with permission from [92]. Royal Society of Chemistry, 2013.

Chen et al. [92] fabricated a high-performance electrochemical SC using Co_3_S_4_ nanosheet arrays on Ni-foam as electrodes, which were prepared by an anion exchange reaction of the Co_3_O_4_ nanosheet arrays. Furthermore, they compared the electrochemical performances of Co_3_S_4_ nanosheet arrays with its corresponding metal oxide analog Co_3_O_4_ nanosheet arrays. Interestingly, the specific capacitance and cycling stability of Co_3_S_4_ nanosheet arrays electrodes were 4.1 times higher than that of Co_3_O_4_ nanosheet arrays, as shown in Figure 5, and achieved a maximum areal capacitance of 1.81 F·cm^−2^ at a current density of 24 mA·cm^−2^. Recently, rGO nanosheets wrapped around Co_3_S_4_ nanoflake electrodes were developed, and their electrochemical performance thoroughly investigated by Patil et al. [93]; the electrode offered a highest specific capacitance of 2314 F·g^−1^ at 2 mV·s^−1^.

(b) CoS

Due to the synergic properties of the metallic and layered characteristics of CoS, it has been widely investigated for use in SC electrodes. Different morphologies of CoS nanostructures have been synthesized by various synthetic routes and have exhibited distinct electrochemical performances. 3-D flower-like hierarchical CoS nanostructure electrodes have been prepared using 6 M KOH solution and employed in SCs, which yielded 586 F·g^−1^ at 1 A·g^−1^ after 1000 cycles [94]. Nevertheless, one-step hydrothermally synthesized two-dimensional (2-D) CoS nanosheet electrodes exhibited superior performance with a higher specific capacitance of around 1314 F·g^−1^ at 3 A·g^−1^ [95]. Later, Wan et al. synthesized and reported the performance of CoS nanotubes for high performance SCs [78], while Justin et al. studied the synthesis of CoS nanospheres using a hydrothermal method and evaluated their applications in SCs [96], and recently, a flower-like CoS hollow sphere electrodes for energy storage devices have been reported [97]. Accordingly, other CoS nanostructures and composites with rGO, titania, and CNT have also been synthesized and studied for SC applications [79,98,99,100].

(c) CoS_1.097_

As with CoS, Wang et al. [101] developed a simple solvothermal method to prepare flower-like 3-D hierarchical CoS_1.097_ and employed it as an SC electrode, which exhibited high specific capacitances of 555 F·g^−1^ and 464 F·g^−1^ at 5 mA·cm^−2^ and 100 mA·cm^−2^, respectively, while 1-D hierarchical CoS_1.097_ on CNT nanostructured electrodes delivered a remarkable specific capacitance of 640 F·g^−1^ at 8 A·g^−1^ after 3000 consecutive CD cycles [102]. Another nanostructure consisting of an ultralong CoS_1.097_ nanotube network provided high specific capacitance, good capacitance retention, and excellent coulombic efficiency, due to its hollow structure and large surface area [103].

(d) CoS_2_

Pyrite-phase cobalt disulfide (CoS_2_) is intrinsically a conductive metal that has been considered as one of the promising materials for wide potential application in SCs [104]. Moreover, it is earth abundant and low cost, and has long-term stability under acidic operating conditions. Furthermore, the thermal stability and Gibbs free energy (−146 kJ·mol^−1^) of CoS_2_ is much higher than that of other metal sulfides, indicating that it has superior capacitive behavior compared to activated carbon positive electrodes for hybrid SCs [105]. As we know, the electrochemical properties of SC electrode materials strongly depend on particle size, shape, and porosity, as well as pore size distribution. Superior electrochemical and pseudocapacitive properties were observed for single phased CoS_2_ ellipsoids, nanoflake thin films, nanowires, octahedrons, and hollow spheres [106,107,108]. The hierarchical mesoporous CoS_2_ electrodes offered a high specific capacitance of 718.7 F·g^−1^ at 1 A·g^−1^ [80], whereas 3-D hollow CoS_2_ nanoframe electrodes fabricated by anion replacement had a maximum capacitance of 568 F·g^−1^ at 0.5 A·g^−1^ [109]. Nevertheless, single component CoS_2_ was intrinsically unstable, which caused several problems, such as relatively low capacitance, poor cycling stability, and rate capability. These could be overcome by an effective synthetic strategy for direct growth of a CoS_2_ active material on a conductive support, which dramatically enhanced the capacitance performance [110]. For instance, CoS_2_-rGO composites which possessed better electrochemical properties than pure individual components have been prepared and investigated recently [111]. Furthermore, a CoS_2_/MoS_2_ on carbon fiber cloth hierarchical electrode exhibited excellent long life cycle stability and achieved a maximum capacitance value of 406 F·g^−1^ [112].

(e) Co_9_S_8_

Various nanostructures of Co_9_S_8_ including nanosheets, nanoneedles, nanospheres, a yolk-shell structure, as well as various heterostructures with CNT and rGO were reported as potential anodes for lithium-ion batteries and dye-sensitized solar cells [113,114,115,116,117,118,119,120]. However, the reports on Co_9_S_8_ nanostructured electrodes leading to SC applications are very scarce. For instance, high purity Co_9_S_8_ thin films on Ni foam have been developed by atomic layer deposition and employed as high-performance SC electrodes which possessed a specific capacitance of 1645 F·g^−1^ at 3 A·g^−1^ [121]. Later Ramachandran et al. [122] suggested a low cost synthetic route for Co_9_S_8_ nanoflake/graphene composite electrodes that offered a maximum specific capacitance of 808 F·g^−1^ at 5 mV·s^−1^ in 6 M KOH electrolyte solution. Mashikwa et al. [123] developed a new type of SC electrode consisting of Co_9_S_8_ nanoparticle clusters embedded in an activated graphene foam structure using a microwave-assisted hydrothermal method; the electrode was capable of delivering a specific capacitance of 1150 F·g^−1^ at 5 mV·s^−1^. Furthermore, 3-D petal-like two-mixed metal sulfide-graphene composite electrodes (Co_9_S_8_/rGO/Ni_3_S_2_/Ni foam) fabricated for high-performance SCs exhibited superior capacitive performance with the high capability (2611.9 F·g^−1^ at 3.9 A·g^−1^), excellent rate capability, and enhanced electrochemical stability with remarkable capacitance retention [124].

### 3.4. Binary Metal Sulfides 

Although many transition metal sulfides have been investigated as electrodes for SCs, binary metal sulfides are quite interesting, due to their higher active redox sites, as well as mechanical and thermal stability compared to that of their corresponding single component counterparts. Most binary metal sulfide nanostructures have been synthesized by applying the Kirkendall effect [125], and recently, various binary metal sulfides have been prepared based on it [82]. In brief, the Kirkendall effect is based on the mutual diffusion process of two metals through an interface so that vacancy diffusion occurs to compensate for the inequality of the material flow and that the initial interface moves. Nevertheless, reports on binary metal sulfides as SC electrode materials are still limited. 

(a) NiCo_2_S_4_

The urchin-like porous NiCo_2_S_4_ nanotubes have been synthesized and employed as pseudocapacitor electrodes with excellent electrochemical performance in the past [107,108]. Later Pu et al. [82] successfully synthesized hollow hexagonal NiCo_2_S_4_ nanoplates, which exhibited a high specific capacitance of 437 F·g^−1^ at 1 A·g^−1^ using 3 M KOH electrolyte solution. CoNi_2_S_4_ electrode materials were successfully fabricated by Du et al. [126]. Self-templating synthesized NiCo_2_S_4_ hollow spheres have shown excellent electrochemical properties, such as an intrinsic electronic conductivity hundreds of times higher than that of its corresponding binary metal oxides [127]; an electrode cell made with it achieved a maximum capacitance of 1263 F·g^−1^ at 2 A·g^−1^ with remarkable rate capability. In the meantime, NiCo_2_S_4_ nanostructures prepared by, for instance, hydrothermal, solvothermal, and polyol methods also exhibited high specific capacitance with fabulous capacitance retention, and were reported as potential pseudocapacitor electrodes for SC applications [81,128,129]. Recently, the NiCo_2_S_4_ on carbon fiber cloth and carbon fiber paper have been investigated, and their electrochemical performances compared to SC applications. NiCo_2_S_4_ carbon fiber paper demonstrated favorable charge-transfer kinetics and fast electron transport compared to NiCo_2_S_4_ carbon fiber cloth, and thus showed superior electrochemical performance compared to its counterpart [130].

(b) Manganese Cobalt Sulfides (MCS)

Great attention has been paid to MCS-based electrodes in the past three years, due to its eco-friendly nature and high redox properties. As with NiCo_2_S_4_, reports on MCS are very few. Previously, Chen et al. [131] synthesized hollow tubular MCS for pseudocapacitor applications. Currently, the ultrathin mesoporous MCS nanosheets have been grown on Ni foam using an electrodeposition technique and characterized for its applications in SCs [132]. Very recently, a high specific capacitance of 1938 F·g^−1^ at 5 A·g^−1^ with long-term cycling stability and capacitance retention have been reported for nano honeycomb-like MCS/3 D-graphene on Ni-foam electrodes [133].

Apart from the above binary metal sulfides, there has only been one report on a 3-D yolk-shell NiGa_2_S_4_ structure confined with nanosheets for high-performance SC applications [134].

### 3.5. Molybdenum Disulfide

In the past decade, MoS_2_ has received a great deal of attention, due to its unique physical and chemical properties and find applications in various fields including electrochromic devices hydrogen storage, catalysis, capacitors, lubricants, and batteries [135,136,137]. In brief, MoS_2_ is a graphene-like 2-D material in which the middle layer of molybdenum is sandwiched between two sulfur layers. All three layers are stacked over each other and held together by weak van der Waals forces [138,139]. In recent times, researchers have focused on the utilization of MoS_2_ to develop high-performance SCs, due to its higher theoretical capacitance (1000 F·g^−1^) than graphite and fast intrinsic ionic conductivity [140,141]. 

Ajayan and co-workers [142] prepared 2-DMoS_2_ film-based micro-SCs by a low-cost spray painting process and subsequent laser printing. The prepared SCs exhibited a better electrochemical performance than graphene-based micro-SCs and delivered a high voltammetric capacitance of 178 F·cm^−3^ with better cycling performance. Later on, several groups have also reported the same range of capacitance values for hydrothermally synthesized MoS_2_ at current density rate of 1 A·g^−1^ [143,144,145,146]. In another typical case, Soon et al. [147] investigated MoS_2_ nanosheets as potential electrodes for SCs, and reported that the SC performance of the electrodes was comparable to that of CNT array electrodes. Recently, chemically deposited MoS_2_ thin films have been synthesized by Pujari et al. [148] using 1 M Na_2_SO_4_ as an electrolyte solution. They showed a specific capacitance value of 180 F·g^−1^ with 82% capacitance retention. Karthikeyan et al. [149] reported MoS_2_-based electrodes prepared using a ball-milling process, and employed them in wire type solid-state SCs. However, in practice, the electronic conductivity of MoS_2_ (10^−5^ Ω^−1^·cm^−1^) is still lower compared to graphene and the specific capacitance of MoS_2_ is very limited when used on its own in SCs [150,151], a deficiency which has been overcome by combining it with other conducting materials (as discussed earlier for metal sulfides).

Huang et al. [152] fabricated a new class of PANI/MoS_2_ composites in which the short rod PANI was anchored onto the surface of MoS_2_. The resultant electrode offered a specific capacitance of 575 F·g^−1^ at 20 mV·s^−1^. The same group extended their research on MoS_2_-graphene nanocomposites and concluded that the capacitance behavior of MoS_2_-graphene composite (243 F·g^−1^) was quite higher than that of bare MoS_2_ (120 F·g^−1^) and bare graphene (35 F·g^−1^) at 1 A·g^−1^, and was comparable with other reported results on MoS_2_-graphene electrodes [83,153,154,155,156,157]. Recently, MoS_2_ decorated laser-induced graphene on polyimide foil-based flexible electrodes [158] have been reported, and showed excellent electrochemical performance. Furthermore, Mandal et al. [159] reported a high specific capacitance value of 253 F·g^−1^ for MoS_2_/rGO composites at 1 A·g^−1^ current density rate, which implies the superiority of MoS_2_ nanocomposites for SCs as high-performance electrodes. Meanwhile, multi-walled CNT/MoS_2_ composites have shown a better specific capacitance and achieved a maximum of (452.7 F·g^−1^) compared to bare MoS_2_ (149.6 F·g^−1^) and bare MWCNT (69.2 F·g^−1^) at a current density rate of 1 A·g^−1^ [160].

Moreover, the utilization of a conducting template along with molybdenum sulfide also improved the surface area and electrochemical performance, and a few classical references are discussed herein. Porous tubular C/MoS_2_ composites using porous aluminum oxide as a template were prepared for the first time by Hu et al. [161], and the prepared electrodes delivered a high capacitance of 210 F·g^−1^ at 1 A·g^−1^ with a very good cycling rate. In another typical case, hydrothermally synthesized C/MoS_2_ having flower-like morphology exhibited a capacitance value of 201.4 F·g^−1^ at 1 A·g^−1^ [162]. Kumuthini et al. [163] prepared MoS_2_@C nanofiber electrodes using an electrospinning process, and achieved high capacitance with 100% life cycle, due to their prominent electrochemical properties with improved stability. In addition, conducting templates like Mo foil, PANI, and PPy have been used along with MoS_2_ as binder-free electrodes for SCs in recent years [164,165,166], but the cycling stability and performances of the MoS_2_-based SCs are not satisfactory and are still a challenge.

### 3.6. Other Transition Metal Sulfides

(a) Bi_2_S_3_

Bi_2_S_3_ is a direct band gap (1.4 eV) layered semiconductor material, and exists mostly in orthorhombic form. In recent years, more attention has been paid to it due to its specific electrical and optical properties, and it has found potentially applicable in the fields of SCs, photocatalysis, sensors, and batteries [167,168]. The important properties of Bi_2_S_3_ leading to SC applications are discussed herein. Rod-like Bi_2_S_3_ micro flowers have been synthesized and characterized for their application in SCs; they provided a maximum specific capacitance of 185.7 F·g^−1^ at 1 A·g^−1^ [169]. Similarly, a recent report on hetero-structured Bi_2_S_3_ nanorod/MoS_2_ nanosheet electrodes showed a specific capacitance of 1258 F·g^−1^ at 10 A·g^−1^ with 92.6% of capacitance retention [85]. Later on, Raut et al. synthesized Bi_2_S_3_ thin films on stainless steel using a successive ionic layer adsorption and reaction (SILAR) method, which improved capacitance performance with long-term cyclability [84].

(b) La_2_S_3_

Due to its stable transition state, the rare earth element lanthanum-based chalcogenides are considered as promising for use in SC electrodes in the current era. Depending on the experimental conditions, lanthanum sulfide exists in different forms, including LaS, La_2_S_3_, and La_3_S_4_, which possess excellent pseudocapacitive behavior and high electronic conductivity similar to other metal sulfides [170]. However, reports on these electrode materials are highly limited, due to their synthetic routes [171]. Most of the reported results on La_2_S_3_ leading to asymmetric SCs have been synthesized using the SILAR method. For instance, Patil et al. [172] prepared La_2_S_3_ thin films on a stainless steel substrate using the SILAR method and studied its electrochemical performance. The resultant electrode delivered a specific capacitance of 256 F·g^−1^ using LiClO_4_/PC electrolyte, while the same La_2_S_3_ electrodes in aqueous electrolytes, such as KOH and Na_2_SO_4_, offered a maximum capacitance of 358 F·g^−1^ at 5 mV·s^−1^ [86]. Later on, they extended their studies to the effect of annealing on these La_2_S_3_ electrodes prepared using chemical bath method, which improved the specific capacitance of the electrodes drastically [173]. Their air-annealed La_2_S_3_ electrodes achieved a maximum of 294 F·g^−1^ at 0.5 mV·s^−1^, which was much higher than bare La_2_S_3_ electrodes. 

(c) WS_2_

WS_2_-based electrode materials are receiving increased attention for applications in SCs, owing to their high specific surface area and adaptable electronic structures. In brief, naturally occurring WS_2_ possesses a hexagonal crystal structure with space group P63/mmc. Each WS_2_ monolayer consists of an individual layer of W atoms with six-fold coordination symmetry, which is then hexagonally packed between two trigonal atomic layers of S atoms [87]. Though it possesses a number of advantages, it did not have electronic conductivity as high as zero band gap graphene, which hampers the direct stand-alone application of WS_2_ in SCs. Quite a few reported results are displayed herein. Ratha and coworkers [87] reported the fabrication of WS_2_/rGO electrodes using a one-step hydrothermal method; these electrodes were capable of delivering 350 F·g^−1^ at 2 mV·S^−1^. Furthermore, the mechanism of WS_2_/rGO nanosheet electrodes was explained by Tu et al. [174]. In the short-term, the charges were stored in the pseudocapacitor via the redox reactions of W^6+^ and W^4+^ on WS_2_, as well as by the O-containing surface functionality on the surface of rGO. It showed excellent specific capacitance with remarkable capacitance retention. Later on, a series of 2-D transition metal carbides (TMCs) , including WS_2_, were investigated, and their strong influence on capacitance studied by Martinez et al. [175]. Bissett et al. [176] analyzed liquid-phase exfoliated WS_2_ electrodes for SCs that offered a maximum specific capacitance of 3.5 F·g^−1^ at 10 mV·S^−1^.

## 4. Transition Metal Selenides

The charge storage mechanism and electrochemical properties of transition metal sulfides for SC applications were discussed in the previous section. This section is purely devoted to a discussion on selenium-based metal chalcogenides for SC applications. Selenium, the nearest neighbor of sulfur in the VI A group, possesses the same valence electrons and oxidation number as sulfur [177]. Hence, the chemical and electrochemical activities of metal selenides almost resemble a metal sulfide, which indicates that the metal selenides may also have promising applications in SCs [178]. Some of the important metal selenides are discussed herein.

### 4.1. Nickel Selenide

Among the transition metal chalcogenides studied, nickel selenides are of particular interest due to their tunable electronic configuration and multiple oxidation states. In addition, they possess resistivity below 10^−3^ Ohm·cm^−1^, due to their paramagnetic nature, which makes them suitable candidates for energy storage devices, especially for SCs [179]. To date, reports on NiSe_2_-based SCs are very limited, due to their highly complicated synthetic routes. The synthesis of NiSe_2_ involves multiple steps, which has led to more expensive capital cost in bulk scale preparations. 

Recently, Wang et al. [180] synthesized truncated cube-like NiSe_2_ single crystals using a simple hydrothermal approach, and deeply studied its electrochemical performance. These electrodes offered a maximum specific capacitance of 1044 F·g^−1^ at 3 A·g^−1^ with an excellent rate capability. Similarly, hexapod-like two-dimensional NiSe_2_ single crystals have been investigated; their electrode delivered a maximum capacitance value of 75 F·g^−1^ at a current density of 1 mA·cm^−2^ with a capacitance retention rate of 94% [181].

### 4.2. Copper Selenide

Inexpensive, semiconducting CuSe has been applied in the fields of optoelectronics, thermoelectrics, and solar cells [182,183]. Due to its variable oxidation states and high electrical conductivity, it is capable of delivering good electrochemical properties. Nevertheless, no reports have hitherto become available on the electrochemical properties of CuSe and only a few studies have been published on CuSe-based SCs. The binder-free pseudo capacitive CuSe_2_/Cu electrodes have been synthesized using a simple hydrothermal method, and the reported electrodes delivered a high specific capacitance of 1037.5 F·g^−1^ at 0.25 mA·cm^−2^ [184]. Moreover, vertically oriented CuSe nanosheet films have recently been developed, and their use in solid-state flexible SCs explored; they exhibited a specific capacitance value of 209 F·g^−1^ [185]. Shinde et al. [186] reported Cu_2_Se nanodentrites as electrodes for high-performance SCs. However, the electrochemical properties of CuSe have not yet been fully identified, and future research is likely to be in the direction of developing high-performance SCs using CuSe electrodes. 

### 4.3. Molybdenum Diselenide

In MoSe_2_, the molybdenum atom is squashed between two selenium atoms by means of strong covalent bonds that characterize the Se-Mo-Se interaction. It has high theoretical capacitance and comprises low-cost and abundant elements. The stacked layers are held together by weak van der Waals forces responsible for ion migration during the CD process. To the best of our knowledge, only very few studies have become available on MoSe_2_ for SC applications. 

Balasingam et al. [187] reported layered MoSe_2_ in a two-electrode configuration using H_2_SO_4_ electrolyte, in which the electrodes possessed very good specific capacitance of 199 F·g^−1^ at 2 mV·s^−1^. The electrode cell’s specific capacitance was increased further by combining MoSe_2_ with rGO to attain a maximum of 211 F·g^−1^ with excellent cyclability. Later on, Haung et al. [188] studied and reported the electrochemical performances of MoSe_2_/graphene composites for SC applications, and the same group recently grew MoSe_2_-based electrodes on Mo-foil and reported their capacitance behavior [189]. Furthermore, low-cost MoSe_2_/MWCNT electrodes have been prepared recently using dip and dry, followed by a chemical bath deposition method [190]. The remarkable performance of the electrodes implies that they would be a potential candidate for high-performance SCs. 

### 4.4. Cobalt Selenides

In recent years, cobalt selenide-based materials have been a new research hot spot in the field of electrochemical SCs, due to their cost-effectiveness and highly reversible nature. There are a variety of compounds including CoSe_2_, CoSe, Co_0.85_Se, Co_3_Se_4_, and Co_2_Se_3_ [191], which have been synthesized using various synthetic routes. To date, very few pleasing results for cobalt selenides and their composites in electrochemical energy storage systems have been published in this regard, which is discussed in this section. 

(a) Co_0.85_Se

Polycrystalline Co_0.85_Se nanotubes having a hollow nanostructure were successfully prepared and investigated by Wang et al. [191]. They also compared the electrochemical and cycling properties of Co_0.85_Se nanotubes with Co_0.85_Se nanoparticles [192], the obtained results indicating that the specific capacitance, cycling stability, and rate capability of Co_0.85_Se nanotubes were superior to those of Co_0.85_Se nanoparticles. Additionally, Co_0.85_Se hollow nanowires have been previously efficaciously synthesized and used as efficient pseudocapacitive electrodes for SCs [193]. Interestingly, Peng et al. [194] employed Co_0.85_Se nanosheets as the positive electrode, and nitrogen-doped porous carbon network as the negative electrode, to fabricate asymmetric SCs, which yielded an energy density of 21.1 W·h·kg^−1^ at a power density of 400 W·kg^−1^ with excellent capacitance retention of 93.8%. Later on, Zhao et al. [195,196] used activated carbon as the negative electrode instead of nitrogen-doped porous carbon network, and reported an energy density of 17.8 W·h·kg^−1^ at a power density of 3.57 kW·kg^−1^ for Co_0.85_Se/AC asymmetric SCs. Meanwhile, Gong et al. [197] replaced Co_0.85_Se nanosheet positive electrodes with Co_0.85_Se nanosheet/Ni-foam, which provided a significant increase in energy density with outstanding cycling stability (39.7 W·h·kg^−1^ at a power density of 789.6 W·kg^−1^). 

(b) CoSe_2_

There has hardly been any investigation into using CoSe_2_ as an electrode material for SCs, and up until now, very few studies have reported using it. However, systematic investigations on the electrochemical performances of metal chalcogenides, such as CoSe_2_ and CoTe_2_, have successfully analyzed and studied their applicability as high performance SCs [198,199]. The CoSe_2_ electrodes delivered a maximum specific capacitance of 951 F·g^−1^ at 5 mV·s^−1^, which is three times higher than that of CoTe_2_. Later on Zhang et al. [200] assembled solid state asymmetric SCs using N-doped CoSe_2_/C as pseudocapacitive electrode whose electrochemical properties have not yet been fully studied. Hence, much effort will be focused on developing these electrodes in the near future.

Other metal selenides such as Ni-Co-Se, WSe_2_, SnSe_2_, and La_2_Se_3_, have been studied for flexible solid-state SC electrodes, but rarely reported [201,202,203,204]. Table 2 represents the electrochemical SC performances of some important metal selenide electrodes.

### 4.5. Binary Metal Selenides

The binary metal selenides are currently highly fascinating, and only a scarce number of reports on their use in SC applications are available, and their electrochemical performance has not yet been fully studied. Nevertheless, some classical examples are presented here. Xia et al. [204] showed that (Ni, Co)_0.85_ Se was able to deliver a highest areal capacitance of 2.33 F·cm^−2^ at 4 mA·cm^−2^ current rate. This super-hydrophilic electrode had metal-like electronic conductivity and offered a maximum conductivity of 1.67 × 10^6^ S·cm^−1^. Similarly, Ni-Co-Se nanowires have shown a high specific capacitance of 86 F·g^−1^ at a current density of 1 A·g^−1^ and excellent cycling stability, with virtually no decrease in capacitance after 2000 continuous CD cycles [201]. More recently, Peng et al. [205,206] prepared two different selenide nanosheet-array electrodes comprising NiSe@MoSe_2_ and Ni_0.85_Se@MoSe_2_, using a hydrothermal method, and studied their use in asymmetric SC applications.

## 5. Summary and Outlook

Currently, the development of SCs as electrochemical energy storage devices is of major importance, with the spotlight on their high power density. A typical SC is composed of electrode material and electrolyte. In an assessment of electrochemical SC devices other than those with a long lifecycle, both their energy and power densities are the two most essential properties. In view of this, current major research focused on SCs is on increasing these characteristics and their life cycle to decrease the cost of the electrode materials. The choice of suitable electrode materials with rational nanostructured designs has resulted in improved electrochemical properties for high performance and cost reduction of SCs. In this review, we conferred recent progress in the advancement of non-metallic oxides, transition metal sulfides and selenides were especially highlighted for SC applications.

The major advantages of electrochemical supercapacitors are high power density (1–10 Kw/kg), lifetime (estimated to be up to 30 years), cycle efficiency (98–100%), operating temperatures (−40 to 70 °C), environmental friendliness, and safety. However, the challenges to be focused on supercapacitor are

(i) Energy density: For practical application, high energy density electrochemical system is required. In view of this, the energy density of electrochemical supercapacitors is less than less than of batteries.
(ii) Cost efficiency: The commonly employed electrode materials such as high porous surface area carbon materials and RuO_2_ are more expensive. Also, the cost of organic electrolytes is far from negligible.
(iii) Self-discharge rate: Electrochemical supercapacitors have high in self discharge rate 10–40%/day.

Nanostructured transition metal chalcogenides have gained huge consideration due to their distinctive chemical stability, electronic properties, and remarkable structure. Among these, transition metal sulfides have been proven to exhibit superior electrochemical performance compared to their bulk counterparts, because of their novel properties associated with decreased size, unique shape, and defective nature. 

Nanoscale structures can effectively improve electrochemical reaction efficiency and utilization of active materials with improved energy and power densities. Extraordinary investigation ought to be done to construct novel electrode materials for SCs, and new ideas and/or design strategies are required in this field. While designing and constructing electrode materials, the researcher ought to take into consideration that they should be abundant, cheap, and eco-friendly for clean technology and potentially be of use in a broad selection of applications.

## Figures and Tables

**Table 1:** Electrochemical supercapacitor (SC) performances of important metal sulfides.

Electrodes	Capacitance (F·g^−1^)	Current Density (A·g^−1^)	Electrolytes	% of Capacity Retention (>1000 Cycles)	Ref.
Ni_3_S_2_	717	2	1 M KOH	91.0	[26]
Ni_3_S_2_@Ni(OH)_2_/3D graphene nanosheet	1037.5	5.1	3 M KOH	99.1	[30]
Ni_3_S_2_/graphene	875.6	1	2 M KOH	93.6	[34]
β-NiS	857.76	2	2 M KOH	99.0	[44]
Ni_3_S_4_@amorphous MoS_2_	1440.9	2	6 M KOH	90.7	[57]
CuS nano-hollow spheres	948	1	6 M KOH	90.0	[51]
CuS@PANI	308.1	0.5	0.1 M Li_2_SO_4_	71.6	[76]
CoS	285	0.5	6 M KOH	99.0	[78]
CoS/graphene	435.7	0.5	6 M KOH	82.3	[79]
CoS_2_ microsphere	718.7	1	6 M KOH	93.0	[80]
NiCo_2_S_4_ nanosphere	1156	1	1 M KOH	82.0	[81]
NiCo_2_S_4_ nanoplates	437	1	3 M KOH	81.0	[82]
MoS_2_	162	0.1	1 M Na_2_SO_4_	93.0	[83]
MoS_2_/graphene	270	0.1	1 M Na_2_SO_4_	89.6	[83]
Bi_2_S_3_	289	(5 mV/s)	1 M Na_2_SO_4_	60.0	[84]
Bi_2_S_3_	1007	1	6 M KOH	92.0	[85]
Bi_2_S_3_/MoS_2_	3040	1	6 M KOH	92.6	[85]
MoS_2_ nanosphere	1565	1	6 M KOH	92.0	[85]
a-La_2_S_3_	256	(5 mV/s)	1M LiClO_4_/PC	85.0	[86]
WS_2_	70	(5 mV/s)	1 M Na_2_SO_4_	-----	[87]
WS_2_/RGO	350	(5 mV/s)	1 M Na_2_SO_4_	99.9	[87]

**Table 2:** Electrochemical SC performances of metal selenides.

Electrodes	Capacitance (F·g^−1^)	Current Density (A·g^−1^)	Electrolytes	% of Capacity Retention (>1000 Cycles)	Ref.
NiSe_2_ single crystal	1044	3	4 M KOH	87.4	[180]
CuSe_2_/Cu	1037.5	(0.25 mA·cm^−2^)	1 M NaOH	104.3	[184]
CuSe nanosheet	209	0.2	1 M Na2SO4	90.0	[185]
Cu_2_Se	688	(5 mV/s)	1 M Na2SO4	86.0	[186]
MoSe_2_ nanosheet	1114.3	1	6 M KOH	104.7	[189]
MoSe_2_/MWCNT	232	1.4	1 M KOH	93.0	[190]
Porous CoSe_2_	951	(5 mV/s)	1 M KOH	52.0	[198]
Co0.85Se nanosheet	1378	1	3 M KOH	95.5	[199]
CoSe_2_/C dodecahedra	726	2	2 M KOH	48.3	[199]
SnSe_2_ nanodisks	168	0.5	6 M KOH	---	[200]
SnSe nanosheets	228	0.5	6 M KOH	---	[200]
Ni-Co-Se	86	1	2 M KOH	100.0	[201]

## Abbreviations

SCs	Supercapacitors
EDLCs	Electric double layer capacitors
CNTs	Carbon nanotubes
MCs	Metal chalcogenides
KOH	Potassium hydroxide
3-D-GN	Three dimensional graphene nanosheet
AC	Activated carbon
rGO	Reduced graphene oxide
MWCNT	Multi-walled carbon nanotubes
PANI	Poly aniline
SILAR	Successive ionic layer adsorption and reaction
LiClO_4_	Lithium perchlorate
PC	Propylene carbonate
TMCs	Transition metal carbides
EC	Ethylene carbonate
CD	Charge-discharge

## References

[1] Theerthagiri J., Senthil R., Senthilkumar B., Polu A.R., Madhavan J., Ashokkumar M. (2017). Recent advances in MoS_2_ nanostructured materials for energy and environmental applications—A review. J. Solid State Chem..

[2] Thiagarajan K., Theerthagiri J., Senthil R., Arunachalam P., Madhavan J., Ghanem M.A. (2017). Synthesis of Ni_3_V_2_O_8_@graphene oxide nanocomposite as an efficient electrode material for supercapacitor applications. J. Solid State Electrochem..

[3] Arunachalam P., Shaddad M.N., Alamoudi A.S., Ghanem M.A., Al-Mayouf A.M. (2017). Microwave-assisted synthesis of Co_3_(PO_4_)_2_ nanospheres for electrocatalytic oxidation of methanol in alkaline media. Catalysts.

[4] Arunachalam P., Ghanem M.A., Al-Mayouf A.M., Al-shalwi M. (2017). Enhanced electrocatalytic performance of mesoporous nickel-cobalt oxide electrode for methanol oxidation in alkaline solution. Mater. Lett..

[5] Theerthagiri J., Sudha R., Premnath K., Arunachalam P., Madhavan J., Al-Mayouf A.M. (2017). Growth of iron diselenide nanorods on graphene oxide nanosheets as advanced electrocatalyst for hydrogen evolution reaction. Int. J. Hydrogen Energy.

[6] Ramesh S., Karuppasamy K., Msolli S., Kim H.-S., Kim H.S., Kim J.-H. (2017). A nanocrystalline structured NiO/MnO_2_@nitrogen-doped graphene oxide hybrid nanocomposite for high performance supercapacitors. New J. Chem..

[7] Thiagarajan K., Theerthagiri J., Senthil R., Madhavan J. (2017). Simple and low cost electrode material based on Ca_2_V_2_O_7_/PANI nanoplatelets for supercapacitor applications. J. Mater. Sci. Mater. Electron..

[8] Zhong C., Deng Y., Hu W., Qiao J., Zhang L., Zhang J. (2015). A review of electrolyte materials and compositions for electrochemical supercapacitors. Chem. Soc. Rev..

[9] Karuppasamy K., Prasanna K., Kim D., Kang Y.H., Rhee H.W. (2017). Headway in rhodanide anion based ternary gel polymer electrolytes (TILGPEs) for applications in rechargeable lithium ion batteries: An efficient route to achieve high electrochemical and cycling performances. RSC Adv..

[10] Karuppasamy K., Rhee H.W., Reddy P.A., Gupta D., Mitu L., Polu A.R., Shajan X.S. (2016). Ionic liquid incorporated nanocomposite polymer electrolytes for rechargeable lithium ion battery: A way to achieve improved electrochemical and interfacial properties. J. Ind. Eng. Chem..

[11] Karuppasamy K., Kim H.-S., Kim D., Vikraman D., Prasanna K., Kathalingam A., Sharma R., Rhee H.W. (2017). An enhanced electrochemical and cycling properties of novel boronic ionic liquid based ternary gel polymer electrolytes for rechargeable Li/LiCoO_2_ cells. Sci. Rep..

[12] Karthikprabhu S., Karuppasamy K., Vikraman D., Prasanna K., Maiyalagan T., Nichelson A., Kathalingam A., Kim H.-S. (2017). Electrochemical performances of LiNi_1−x_Mn_x_PO_4_ (x = 0.05–0.2) olivine cathode materials for high voltage rechargeable lithium ion batteries. Appl. Surf. Sci..

[13] Borenstein A., Hanna O., Attias R., Luski S., Brousse T., Aurbach D. (2017). Carbon-based composite materials for supercapacitor electrodes: A review. J. Mater. Chem. A.

[14] Zhang Y., Yu S., Lou G., Shen Y., Chen H., Shen Z., Zhao S., Zhang J., Chai S., Zou Q. (2017). Review of macroporous materials as electrochemical supercapacitor electrodes. J. Mater. Sci..

[15] Ke Q., Wang J. (2016). Graphene-based materials for supercapacitor electrodes—A review. J. Materiomics.

[16] Vadivel S., Naveen A., Theerthagiri J., Madhavan J., Priya T.S., Balasubramanian N. (2016). Solvothermal synthesis of BiPO_4_ nanorods/MWCNT (1D-1D) composite for photocatalyst and supercapacitor applications. Ceram. Int..

[17] Zhang X., Wang X., Jiang L., Wu H., Wu C., Su J. (2012). Effect of aqueous electrolytes on the electrochemical behaviors of supercapacitors based on hierarchically porous carbons. J. Power Sources.

[18] Qiao J., Zhong C., Deng Y., Hu W., Sun D., Han X., Zhang J. (2016). Electrolytes for Electrochemical Supercapacitors.

[19] Theerthagiri J., Thiagarajan K., Senthilkumar B., Khan Z., Senthil R.A., Arunachalam P., Madhavan J., Ashokkumar M. (2017). Synthesis of hierarchical cobalt phosphate nanoflakes and their enhanced electrochemical performances for supercapacitor applications. ChemistrySelect.

[20] Senthilkumar B., Khan Z., Park S., Kim K., Ko H., Kim Y. (2015). Highly porous graphitic carbon and Ni_2_P_2_O_7_ for a high performance aqueous hybrid supercapacitor. J. Mater. Chem. A.

[21] Simon P., Gogotsi Y. (2008). Materials for electrochemical capacitors. Nat. Mater..

[22] Wang G., Zhang L., Zhang J. (2012). A review of electrode materials for electrochemical supercapacitors. Chem. Soc. Rev..

[23] Kötz R., Carlen M. (2000). Principles and applications of electrochemical capacitors. Electrochim. Acta.

[24] Pang H., Wei C., Li X., Li G., Ma Y., Li S., Chen J., Zhang J. (2014). Microwave-assisted synthesis of NiS_2_ nanostructures for supercapacitors and cocatalytic enhancing photocatalytic H_2_ production. Sci. Rep..

[25] Peng S., Li L., Tan H., Cai R., Shi W., Li C., Mhaisalkar S.G., Srinivasan M., Ramakrishna S., Yan Q. (2014). MS_2_ (M = Co and Ni) hollow spheres with tunable interiors for high-performance supercapacitors and photovoltaics. Adv. Funct. Mater..

[26] Chou S.-W., Lin J.-Y. (2013). Cathodic deposition of flaky nickel sulfide nanostructure as an electroactive material for high-performance supercapacitors. J. Electrochem. Soc..

[27] Krishnamoorthy K., Veerasubramani G.K., Radhakrishnan S., Kim S.J. (2014). One pot hydrothermal growth of hierarchical nanostructured Ni_3_S_2_ on Ni foam for supercapacitor application. Chem. Eng. J..

[28] Zhang Z., Huang Z., Ren L., Shen Y., Qi X., Zhong J. (2014). One-pot synthesis of hierarchically nanostructured Ni_3_S_2_ dendrites as active materials for supercapacitors. Electrochim. Acta.

[29] Huo H., Zhao Y., Xu C. (2014). 3D Ni_3_S_2_ nanosheet arrays supported on Ni foam for high-performance supercapacitor and non-enzymatic glucose detection. J. Mater. Chem. A.

[30] Zhou W., Cao X., Zeng Z., Shi W., Zhu Y., Yan Q., Liu H., Wang J., Zhang H. (2013). One-step synthesis of Ni_3_S_2_ nanorod@Ni(OH)_2_ nanosheet core–shell nanostructures on a three-dimensional graphene network for high-performance supercapacitors. Energy Environ. Sci..

[31] Shehzad K., Xu Y., Gao C., Duan X. (2016). Three-dimensional macro-structures of two-dimensional nanomaterials. Chem. Soc. Rev..

[32] Zhu T., Wu H.B., Wang Y., Xu R., Lou X.W.D. (2012). Formation of 1D hierarchical structures composed of Ni_3_S_2_ nanosheets on CNTs backbone for supercapacitors and photocatalytic H_2_ production. Adv. Energy Mater..

[33] Pan S., Zhu J., Liu X. (2013). Preparation, electrochemical properties, and adsorption kinetics of Ni_3_S_2_/graphene nanocomposites using alkyldithiocarbonatio complexes of nickel(ii) as single-source precursors. New J. Chem..

[34] Ou X., Gan L., Luo Z. (2014). Graphene-templated growth of hollow Ni_3_S_2_ nanoparticles with enhanced pseudocapacitive performance. J. Mater. Chem. A.

[35] Ou X., Luo Z. (2016). One-step synthesis of Ni_3_S_2_ nanoplatelets on graphene for high performance supercapacitors. RSC Adv..

[36] Xing Z., Chu Q., Ren X., Tian J., Asiri A.M., Alamry K.A., Al-Youbi A.O., Sun X. (2013). Biomolecule-assisted synthesis of nickel sulfides/reduced graphene oxide nanocomposites as electrode materials for supercapacitors. Electrochem. Commun..

[37] Zhang Z., Zhao C., Min S., Qian X. (2014). A facile one-step route to RGO/Ni_3_S_2_ for high-performance supercapacitors. Electrochim. Acta.

[38] Zang X., Dai Z., Yang J., Zhang Y., Huang W., Dong X. (2016). Template-assisted synthesis of nickel sulfide nanowires: Tuning the compositions for supercapacitors with improved electrochemical stability. ACS Appl. Mater. Interfaces.

[39] Chen J.S., Guan C., Gui Y., Blackwood D.J. (2016). Rational design of self-supported Ni_3_S_2_ nanosheets array for advanced asymmetric supercapacitor with a superior energy density. ACS Appl. Mater. Interfaces.

[40] Stender C.L., Odom T.W. (2007). Chemical nanofabrication: A general route to surface-patterned and free-standing transition metal chalcogenide nanostructures. J. Mater. Chem..

[41] Yang S.-L., Yao H.-B., Gao M.-R., Yu S.-H. (2009). Monodisperse cubic pyrite NiS_2_ dodecahedrons and microspheres synthesized by a solvothermal process in a mixed solvent: Thermal stability and magnetic properties. CrystEngComm.

[42] Ruan Y., Jiang J., Wan H., Ji X., Miao L., Peng L., Zhang B., Lv L., Liu J. (2016). Rapid self-assembly of porous square rod-like nickel persulfide via a facile solution method for high-performance supercapacitors. J. Power Sources.

[43] Dai S., Zhao B., Qu C., Chen D., Dang D., Song B., Fu J., Hu C., Wong C.-P., Liu M. (2017). Controlled synthesis of three-phase Ni_x_S_y_/rGO nanoflake electrodes for hybrid supercapacitors with high energy and power density. Nano Energy.

[44] Xiong X., Zhao B., Ding D., Chen D., Yang C., Lei Y., Liu M. (2016). One-step synthesis of architectural Ni_3_S_2_ nanosheet-on-nanorods array for use as high-performance electrodes for supercapacitors. NPG Asia Mater..

[45] Qu C., Zhang L., Meng W., Liang Z., Zhu B., Dang D., Dai S., Zhao B., Tabassum H., Gao S. (2018). MOF-derived α-NiS nanorods on graphene as an electrode for high-energy-density supercapacitors. J. Mater. Chem. A.

[46] Zhang Y., Zuo L., Zhang L., Yan J., Lu H., Fan W., Liu T. (2016). Immobilization of NiS nanoparticles on n-doped carbon fiber aerogels as advanced electrode materials for supercapacitors. Nano Res..

[47] Yang J., Duan X., Qin Q., Zheng W. (2013). Solvothermal synthesis of hierarchical flower-like β-NiS with excellent electrochemical performance for supercapacitors. J. Mater. Chem. A.

[48] Wang Z., Nan C., Wang D., Li Y. (2014). Fabrication of 1D nickel sulfide nanocrystals with high capacitances and remarkable durability. RSC Adv..

[49] Wei C., Cheng C., Zhao J., Wang Y., Cheng Y., Xu Y., Du W., Pang H. (2015). NiS hollow spheres for high-performance supercapacitors and non-enzymatic glucose sensors. Chem. Asian J..

[50] Sun C., Ma M., Yang J., Zhang Y., Chen P., Huang W., Dong X. (2014). Phase-controlled synthesis of α-NiS nanoparticles confined in carbon nanorods for high performance supercapacitors. Sci. Rep..

[51] Yu L., Yang B., Liu Q., Liu J., Wang X., Song D., Wang J., Jing X. (2015). Interconnected NiS nanosheets supported by nickel foam: Soaking fabrication and supercapacitors application. J. Electroanal. Chem..

[52] Tran V.C., Sahoo S., Shim J.-J. (2018). Room-temperature synthesis of NiS hollow spheres on nickel foam for high-performance supercapacitor electrodes. Mater. Lett..

[53] Li Z., Yu X., Gu A., Tang H., Wang L., Lou Z. (2017). Anion exchange strategy to synthesis of porous NiS hexagonal nanoplates for supercapacitors. Nanotechnology.

[54] Jothi P.R., Salunkhe R.R., Pramanik M., Kannan S., Yamauchi Y. (2016). Surfactant-assisted synthesis of nanoporous nickel sulfide flakes and their hybridization with reduced graphene oxides for supercapacitor applications. RSC Adv..

[55] LiáZhang L. (2015). Rigid three-dimensional Ni_3_S_4_ nanosheet frames: Controlled synthesis and their enhanced electrochemical performance. RSC Adv..

[56] Huang F., Yan A., Sui Y., Wei F., Qi J., Meng Q., He Y. (2017). One-step hydrothermal synthesis of Ni_3_S_4_@MoS_2_ nanosheet on carbon fiber paper as a binder-free anode for supercapacitor. J. Mater. Sci. Mater. Electron..

[57] Zhang Y., Sun W., Rui X., Li B., Tan H.T., Guo G., Madhavi S., Zong Y., Yan Q. (2015). One-pot synthesis of tunable crystalline Ni_3_S_4_@amorphous MoS_2_ core/shell nanospheres for high-performance supercapacitors. Small.

[58] Luo W., Zhang G., Cui Y., Sun Y., Qin Q., Zhang J., Zheng W. (2017). One-step extended strategy for the ionic liquid-assisted synthesis of Ni_3_S_4_–MoS_2_ heterojunction electrodes for supercapacitors. J. Mater. Chem. A.

[59] Li S., Chen T., Wen J., Gui P., Fang G. (2017). In situ grown Ni_9_S_8_ nanorod/O-MoS_2_ nanosheet nanocomposite on carbon cloth as a free binder supercapacitor electrode and hydrogen evolution catalyst. Nanotechnology.

[60] Quintana-Ramirez P.V., Arenas-Arrocena M.C., Santos-Cruz J., Vega-González M., Martínez-Alvarez O., Castaño-Meneses V.M., Acosta-Torres L.S., de la Fuente-Hernández J. (2014). Growth evolution and phase transition from chalcocite to digenite in nanocrystalline copper sulfide: Morphological, optical and electrical properties. Beilstein J. Nanotechnol..

[61] Evans H.T. (1981). Copper coordination in low chalcocite and djurleite and other copper-rich sulfides. Am. Mineral..

[62] Zhu T., Xia B., Zhou L., Lou X.W.D. (2012). Arrays of ultrafine CuS nanoneedles supported on a CNT backbone for application in supercapacitors. J. Mater. Chem..

[63] Ghezelbash A., Korgel B.A. (2005). Nickel sulfide and copper sulfide nanocrystal synthesis and polymorphism. Langmuir.

[64] Huang K.-J., Zhang J.-Z., Fan Y. (2015). One-step solvothermal synthesis of different morphologies CuS nanosheets compared as supercapacitor electrode materials. J. Alloys Compd..

[65] Krishnamoorthy K., Veerasubramani G.K., Rao A.N., Kim S.J. (2014). One-pot hydrothermal synthesis, characterization and electrochemical properties of CuS nanoparticles towards supercapacitor applications. Mater. Res. Express.

[66] Heydari H., Moosavifard S.E., Elyasi S., Shahraki M. (2017). Nanoporous CuS nano-hollow spheres as advanced material for high-performance supercapacitors. Appl. Surf. Sci..

[67] Lee Y.-W., Kim B.-S., Hong J., Choi H., Jang H.-S., Hou B., Pak S., Lee J., Lee S.-H., Morris S.M. (2017). Hierarchically assembled tubular shell-core-shell heterostructure of hybrid transition metal chalcogenides for high-performance supercapacitors with ultrahigh cyclability. Nano Energy.

[68] Peng H., Ma G., Mu J., Sun K., Lei Z. (2014). Controllable synthesis of CuS with hierarchical structures via a surfactant-free method for high-performance supercapacitors. Mater. Lett..

[69] Krishnamoorthy K., Veerasubramani G.K., Radhakrishnan S., Kim S.J. (2015). Preparation of copper sulfide nanoparticles by sonochemical method and study on their electrochemical properties. J. Nanosci. Nanotechnol..

[70] Huang K.-J., Zhang J.-Z., Liu Y., Liu Y.-M. (2015). Synthesis of reduced graphene oxide wrapped-copper sulfide hollow spheres as electrode material for supercapacitor. Int. J. Hydrogen Energy.

[71] Huang K.-J., Zhang J.-Z., Xing K. (2014). One-step synthesis of layered CuS/multi-walled carbon nanotube nanocomposites for supercapacitor electrode material with ultrahigh specific capacitance. Electrochim. Acta.

[72] Lu Y., Liu X., Wang W., Cheng J., Yan H., Tang C., Kim J.-K., Luo Y. (2015). Hierarchical, porous CuS microspheres integrated with carbon nanotubes for high-performance supercapacitors. Sci. Rep..

[73] Peng H., Ma G., Sun K., Mu J., Wang H., Lei Z. (2014). High-performance supercapacitor based on multi-structural CuS@polypyrrole composites prepared by in situ oxidative polymerization. J. Mater. Chem. A.

[74] Lee Y.-W., Kim B.-S., Hong J., Lee J., Pak S., Jang H.-S., Whang D., Cha S., Sohn J.I., Kim J.M. (2016). A pseudo-capacitive chalcogenide-based electrode with dense 1-dimensional nanoarrays for enhanced energy density in asymmetric supercapacitors. J. Mater. Chem. A.

[75] Huang K.-J., Zhang J.-Z., Jia Y.-L., Xing K., Liu Y.-M. (2015). Acetylene black incorporated layered copper sulfide nanosheets for high-performance supercapacitor. J. Alloys Compd..

[76] Chen C., Zhang Q., Peng C. (2017). Facile synthesis of core-shell structured CuS@PANI microspheres and electrochemical capacitance investigations. Polym. Polym. Compos..

[77] Gopi C.V.M., Ravi S., Rao S.S., Reddy A.E., Kim H.-J. (2017). Carbon nanotube/metal-sulfide composite flexible electrodes for high-performance quantum dot-sensitized solar cells and supercapacitors. Sci. Rep..

[78] Wan H., Ji X., Jiang J., Yu J., Miao L., Zhang L., Bie S., Chen H., Ruan Y. (2013). Hydrothermal synthesis of cobalt sulfide nanotubes: The size control and its application in supercapacitors. J. Power Sources.

[79] Meng X., Sun H., Zhu J., Bi H., Han Q., Liu X., Wang X. (2016). Graphene-based cobalt sulfide composite hydrogel with enhanced electrochemical properties for supercapacitors. New J. Chem..

[80] Xing J.-C., Zhu Y.-L., Li M.-Y., Jiao Q.-J. (2014). Hierarchical mesoporous CoS_2_ microspheres: Morphology-controlled synthesis and their superior pseudocapacitive properties. Electrochim. Acta.

[81] Zhang Y., Sui Y., Qi J., Hou P., Wei F., He Y., Meng Q., Sun Z. (2017). Facile synthesis of NiCo_2_S_4_ spheres with granular core used as supercapacitor electrode materials. J. Mater. Sci. Mater. Electron..

[82] Pu J., Cui F., Chu S., Wang T., Sheng E., Wang Z. (2013). Preparation and electrochemical characterization of hollow hexagonal NiCo_2_S_4_ nanoplates as pseudocapacitor materials. ACS Sustain. Chem. Eng..

[83] Thangappan R., Kalaiselvam S., Elayaperumal A., Jayavel R., Arivanandhan M., Karthikeyan R., Hayakawa Y. (2016). Graphene decorated with MoS_2_ nanosheets: A synergetic energy storage composite electrode for supercapacitor applications. Dalton Trans..

[84] Raut S.S., Dhobale J.A., Sankapal B.R. (2017). Silar deposited Bi_2_S_3_ thin film towards electrochemical supercapacitor. Phys. E Low-Dimens. Syst. Nanostruct..

[85] Fang L., Qiu Y., Zhai T., Wang F., Lan M., Huang K., Jing Q. (2017). Flower-like nanoarchitecture assembled from Bi_2_S_3_ nanorod/MoS_2_ nanosheet heterostructures for high-performance supercapacitor electrodes. Colloids Surf. A Physicochem. Eng. Aspects.

[86] Patil S., Kumbhar V., Patil B., Bulakhe R., Lokhande C. (2014). Chemical synthesis of α-La_2_S_3_ thin film as an advanced electrode material for supercapacitor application. J. Alloys Compd..

[87] Ratha S., Rout C.S. (2013). Supercapacitor electrodes based on layered tungsten disulfide-reduced graphene oxide hybrids synthesized by a facile hydrothermal method. ACS Appl. Mater. Interfaces.

[88] Otero-Leal M., Rivadulla F., Rivas J. (2008). The magnetic phase transition of CoS_2−x_Se_x_. IEEE Trans. Magn..

[89] Sadjadi M., Pourahmad A., Sohrabnezhad S., Zare K. (2007). Formation of NiS and CoS semiconductor nanoparticles inside mordenite-type zeolite. Mater. Lett..

[90] Behret H., Binder H., Sandstede G. (1975). Electrocatalytic oxygen reduction with thiospinels and other sulphides of transition metals. Electrochim. Acta.

[91] Sohrabnezhad S., Pourahmad A., Radaee E. (2009). Photocatalytic degradation of basic blue 9 by CoS nanoparticles supported on ALMCM-41 material as a catalyst. J. Hazard. Mater..

[92] Chen Q., Li H., Cai C., Yang S., Huang K., Wei X., Zhong J. (2013). In situ shape and phase transformation synthesis of Co_3_S_4_ nanosheet arrays for high-performance electrochemical supercapacitors. RSC Adv..

[93] Patil S., Kim J., Lee D. (2017). Graphene-nanosheet wrapped cobalt sulphide as a binder free hybrid electrode for asymmetric solid-state supercapacitor. J. Power Sources.

[94] Luo F., Li J., Yuan H., Xiao D. (2014). Rapid synthesis of three-dimensional flower-like cobalt sulfide hierarchitectures by microwave assisted heating method for high-performance supercapacitors. Electrochim. Acta.

[95] Huang K.-J., Zhang J.-Z., Shi G.-W., Liu Y.-M. (2014). One-step hydrothermal synthesis of two-dimensional cobalt sulfide for high-performance supercapacitors. Mater. Lett..

[96] Justin P., Rao G.R. (2010). CoS spheres for high-rate electrochemical capacitive energy storage application. Int. J. Hydrogen Energy.

[97] Wang Q., Jiao L., Han Y., Du H., Peng W., Huan Q., Song D., Si Y., Wang Y., Yuan H. (2011). CoS_2_ hollow spheres: Fabrication and their application in lithium-ion batteries. J. Phys. Chem. C.

[98] Ranaweera C., Wang Z., Alqurashi E., Kahol P., Dvornic P., Gupta B.K., Ramasamy K., Mohite A.D., Gupta G., Gupta R.K. (2016). Highly stable hollow bifunctional cobalt sulfides for flexible supercapacitors and hydrogen evolution. J. Mater. Chem. A.

[99] Subramani K., Sudhan N., Divya R., Sathish M. (2017). All-solid-state asymmetric supercapacitors based on cobalt hexacyanoferrate-derived CoS and activated carbon. RSC Adv..

[100] Ray R.S., Sarma B., Jurovitzki A.L., Misra M. (2015). Fabrication and characterization of titania nanotube/cobalt sulfide supercapacitor electrode in various electrolytes. Chem. Eng. J..

[101] Wang Q., Jiao L., Du H., Yang J., Huan Q., Peng W., Si Y., Wang Y., Yuan H. (2011). Facile synthesis and superior supercapacitor performances of three-dimensional cobalt sulfide hierarchitectures. CrystEngComm.

[102] Lin J.-Y., Tai S.-Y., Chou S.-W. (2013). Bifunctional one-dimensional hierarchical nanostructures composed of cobalt sulfide nanoclusters on carbon nanotubes backbone for dye-sensitized solar cells and supercapacitors. J. Phys. Chem. C.

[103] Liu S., Mao C., Niu Y., Yi F., Hou J., Lu S., Jiang J., Xu M., Li C. (2015). Facile synthesis of novel networked ultralong cobalt sulfide nanotubes and its application in supercapacitors. ACS Appl. Mater. Interfaces.

[104] Liu G., Wang B., Wang L., Yuan Y., Wang D. (2016). A facile hydrothermal synthesis of a reduced graphene oxide modified cobalt disulfide composite electrode for high-performance supercapacitors. RSC Adv..

[105] Pujari R., Lokhande A., Kim J., Lokhande C. (2016). Bath temperature controlled phase stability of hierarchical nanoflakes CoS_2_ thin films for supercapacitor application. RSC Adv..

[106] Zhang L., Wu H.B., Lou X.W.D. (2012). Unusual CoS_2_ ellipsoids with anisotropic tube-like cavities and their application in supercapacitors. Chem. Commun..

[107] Ren R., Faber M.S., Dziedzic R., Wen Z., Jin S., Mao S., Chen J. (2015). Metallic CoS_2_ nanowire electrodes for high cycling performance supercapacitors. Nanotechnology.

[108] Xing J.-C., Zhu Y.-L., Zhou Q.-W., Zheng X.-D., Jiao Q.-J. (2014). Fabrication and shape evolution of CoS_2_ octahedrons for application in supercapacitors. Electrochim. Acta.

[109] Zeng X., Yang B., Li X., Yu R. (2017). Three-dimensional hollow CoS_2_ nanoframes fabricated by anion replacement and their enhanced pseudocapacitive performances. Electrochim. Acta.

[110] Wei T.Y., Chen C.H., Chien H.C., Lu S.Y., Hu C.C. (2010). A cost-effective supercapacitor material of ultrahigh specific capacitances: Spinel nickel cobaltite aerogels from an epoxide-driven sol–gel process. Adv. Mater..

[111] Tang J., Shen J., Li N., Ye M. (2014). A free template strategy for the synthesis of CoS_2_-reduced graphene oxide nanocomposite with enhanced electrode performance for supercapacitors. Ceram. Int..

[112] Su C., Xiang J., Wen F., Song L., Mu C., Xu D., Hao C., Liu Z. (2016). Microwave synthesized three-dimensional hierarchical nanostructure CoS_2_/MoS_2_ growth on carbon fiber cloth: A bifunctional electrode for hydrogen evolution reaction and supercapacitor. Electrochim. Acta.

[113] Ko Y.N., Choi S.H., Park S.B., Kang Y.C. (2014). Preparation of yolk-shell and filled Co_9_S_8_ microspheres and comparison of their electrochemical properties. Chem. Asian J..

[114] Zhou Y., Yan D., Xu H., Liu S., Yang J., Qian Y. (2015). Multiwalled carbon nanotube@a-C@Co_9_S_8_ nanocomposites: A high-capacity and long-life anode material for advanced lithium ion batteries. Nanoscale.

[115] Su Q., Du G., Zhang J., Zhong Y., Xu B., Yang Y., Neupane S., Li W. (2014). In situ transmission electron microscopy observation of electrochemical sodiation of individual Co_9_S_8_-filled carbon nanotubes. ACS Nano.

[116] Zhou Y., Yan D., Xu H., Feng J., Jiang X., Yue J., Yang J., Qian Y. (2015). Hollow nanospheres of mesoporous Co_9_S_8_ as a high-capacity and long-life anode for advanced lithium ion batteries. Nano Energy.

[117] Zhang X., Liu Q., Meng L., Wang H., Bi W., Peng Y., Yao T., Wei S., Xie Y. (2013). In-plane coassembly route to atomically thick inorganic–organic hybrid nanosheets. ACS Nano.

[118] Chen C., Ye M., Zhang N., Wen X., Zheng D., Lin C. (2015). Preparation of hollow Co_9_S_8_ nanoneedle arrays as effective counter electrodes for quantum dot-sensitized solar cells. J. Mater. Chem. A.

[119] Zhao B., Chen D., Xiong X., Song B., Hu R., Zhang Q., Rainwater B.H., Waller G.H., Zhen D., Ding Y. (2017). A high-energy, long cycle-life hybrid supercapacitor based on graphene composite electrodes. Energy Storage Mater..

[120] Deng X., Zhao B., Zhu L., Shao Z. (2015). Molten salt synthesis of nitrogen-doped carbon with hierarchical pore structures for use as high-performance electrodes in supercapacitors. Carbon.

[121] Li H., Gao Y., Shao Y., Su Y., Wang X. (2015). Vapor-phase atomic layer deposition of Co_9_S_8_ and its application for supercapacitors. Nano Lett..

[122] Ramachandran R., Saranya M., Santhosh C., Velmurugan V., Raghupathy B.P., Jeong S.K., Grace A.N. (2014). Co_9_S_8_ nanoflakes on graphene (Co_9_S_8_/G) nanocomposites for high performance supercapacitors. RSC Adv..

[123] Masikhwa T.M., Madito M.J., Bello A., Lekitima J., Manyala N. (2017). Microwave-assisted synthesis of cobalt sulphide nanoparticle clusters on activated graphene foam for electrochemical supercapacitors. RSC Adv..

[124] Zhang Z., Wang Q., Zhao C., Min S., Qian X. (2015). One-step hydrothermal synthesis of 3D petal-like Co_9_S_8_/RGO/Ni_3_S_2_ composite on nickel foam for high-performance supercapacitors. ACS Appl. Mater. Interfaces.

[125] Wang Z., Pan L., Hu H., Zhao S. (2010). Co_9_S_8_ nanotubes synthesized on the basis of nanoscale kirkendall effect and their magnetic and electrochemical properties. CrystEngComm.

[126] Du W., Zhu Z., Wang Y., Liu J., Yang W., Qian X., Pang H. (2014). One-step synthesis of CoNi_2_S_4_ nanoparticles for supercapacitor electrodes. RSC Adv..

[127] Xia C., Alshareef H.N. (2015). Self-templating scheme for the synthesis of nanostructured transition-metal chalcogenide electrodes for capacitive energy storage. Chem. Mater..

[128] Hua H., Liu S., Chen Z., Bao R., Shi Y., Hou L., Pang G., Hui K.N., Zhang X., Yuan C. (2016). Self-sacrifice template formation of hollow hetero-Ni_7_S_6_/Co_3_S_4_ nanoboxes with intriguing pseudo-capacitance for high-performance electrochemical capacitors. Sci. Rep..

[129] Wu Z., Pu X., Ji X., Zhu Y., Jing M., Chen Q., Jiao F. (2015). High energy density asymmetric supercapacitors from mesoporous NiCo_2_S_4_ nanosheets. Electrochim. Acta.

[130] Sun M., Tie J., Cheng G., Lin T., Peng S., Deng F., Ye F., Yu L. (2015). In situ growth of burl-like nickel cobalt sulfide on carbon fibers as high-performance supercapacitors. J. Mater. Chem. A.

[131] Chen Y.M., Li Z., Lou X.W.D. (2015). General formation of M*x*Co_3−_*x*S_4_ (M = Ni, Mn, Zn) hollow tubular structures for hybrid supercapacitors. Angew. Chem..

[132] Sahoo S., Rout C.S. (2016). Facile electrochemical synthesis of porous manganese-cobalt-sulfide based ternary transition metal sulfide nanosheets architectures for high performance energy storage applications. Electrochim. Acta.

[133] Yu M., Li X., Ma Y., Liu R., Liu J., Li S. (2017). Nanohoneycomb-like manganese cobalt sulfide/three dimensional graphene-nickel foam hybid electrodes for high-rate capability supercapacitors. Appl. Surf. Sci..

[134] Liu S., Kim K.H., Yun J.M., Kundu A., Sankar K.V., Patil U.M., Ray C., Jun S.C. (2017). 3D yolk–shell NiGa_2_S_4_ microspheres confined with nanosheets for high performance supercapacitors. J. Mater. Chem. A.

[135] Chen J., Li S.-L., Xu Q., Tanaka K. (2002). Synthesis of open-ended MoS_2_ nanotubes and the application as the catalyst of methanation. Chem. Commun..

[136] Chen J., Kuriyama N., Yuan H., Takeshita H.T., Sakai T. (2001). Electrochemical hydrogen storage in MoS_2_ nanotubes. J. Am. Chem. Soc..

[137] Ding S., Chen J.S., Lou X.W.D. (2011). Glucose-assisted growth of MoS_2_ nanosheets on CNT backbone for improved lithium storage properties. Chem. Eur. J..

[138] Ma G., Peng H., Mu J., Huang H., Zhou X., Lei Z. (2013). In situ intercalative polymerization of pyrrole in graphene analogue of MoS_2_ as advanced electrode material in supercapacitor. J. Power Sources.

[139] Zhang G., Liu H., Qu J., Li J. (2016). Two-dimensional layered MoS_2_: Rational design, properties and electrochemical applications. Energy Environ. Sci..

[140] Zheng N., Bu X., Feng P. (2003). Synthetic design of crystalline inorganic chalcogenides exhibiting fast-ion conductivity. Nature.

[141] Wang H., Feng H., Li J. (2014). Graphene and graphene-like layered transition metal dichalcogenides in energy conversion and storage. Small.

[142] Cao L., Yang S., Gao W., Liu Z., Gong Y., Ma L., Shi G., Lei S., Zhang Y., Zhang S. (2013). Direct laser-patterned micro-supercapacitors from paintable MoS_2_ films. Small.

[143] Ramadoss A., Kim T., Kim G.-S., Kim S.J. (2014). Enhanced activity of a hydrothermally synthesized mesoporous MoS_2_ nanostructure for high performance supercapacitor applications. New J. Chem..

[144] Krishnamoorthy K., Veerasubramani G.K., Radhakrishnan S., Kim S.J. (2014). Supercapacitive properties of hydrothermally synthesized sphere like MoS_2_ nanostructures. Mater. Res. Bull..

[145] Ilanchezhiyan P., Kumar G.M., Kang T. (2015). Electrochemical studies of spherically clustered MoS_2_ nanostructures for electrode applications. J. Alloys Compd..

[146] Acerce M., Voiry D., Chhowalla M. (2015). Metallic 1T phase MoS_2_ nanosheets as supercapacitor electrode materials. Nat. Nanotechnol..

[147] Soon J.M., Loh K.P. (2007). Electrochemical double-layer capacitance of MoS_2_ nanowall films. Electrochem. Solid-State Lett..

[148] Pujari R., Lokhande A., Shelke A., Kim J., Lokhande C. (2017). Chemically deposited nano grain composed MoS_2_ thin films for supercapacitor application. J. Colloid Interface Sci..

[149] Krishnamoorthy K., Pazhamalai P., Veerasubramani G.K., Kim S.J. (2016). Mechanically delaminated few layered MoS_2_ nanosheets based high performance wire type solid-state symmetric supercapacitors. J. Power Sources.

[150] Huang K.-J., Wang L., Liu Y.-J., Wang H.-B., Liu Y.-M., Wang L.-L. (2013). Synthesis of polyaniline/2-dimensional graphene analog MoS_2_ composites for high-performance supercapacitor. Electrochim. Acta.

[151] Falola B.D., Wiltowski T., Suni I.I. (2016). Electrodeposition of MoS_2_ for charge storage in electrochemical supercapacitors. J. Electrochem. Soc..

[152] Huang K.-J., Wang L., Liu Y.-J., Liu Y.-M., Wang H.-B., Gan T., Wang L.-L. (2013). Layered MoS_2_–graphene composites for supercapacitor applications with enhanced capacitive performance. Int. J. Hydrogen Energy.

[153] Bissett M.A., Kinloch I.A., Dryfe R.A. (2015). Characterization of MoS_2_–graphene composites for high-performance coin cell supercapacitors. ACS Appl. Mater. Interfaces.

[154] Gopalakrishnan K., Pramoda K., Maitra U., Mahima U., Shah M., Rao C. (2015). Performance of MoS_2_-reduced graphene oxide nanocomposites in supercapacitors and in oxygen reduction reaction. Nanomater. Energy.

[155] Xie B., Chen Y., Yu M., Sun T., Lu L., Xie T., Zhang Y., Wu Y. (2016). Hydrothermal synthesis of layered molybdenum sulfide/n-doped graphene hybrid with enhanced supercapacitor performance. Carbon.

[156] Masikhwa T.M., Madito M.J., Bello A., Dangbegnon J.K., Manyala N. (2017). High performance asymmetric supercapacitor based on molybdenum disulphide/graphene foam and activated carbon from expanded graphite. J. Colloid Interface Sci..

[157] Patil U.M., Nam M.S., Kang S., Sohn J.S., Sim H.B., Kang S., Jun S.C. (2016). Fabrication of ultra-high energy and power asymmetric supercapacitors based on hybrid 2D MoS_2_/graphene oxide composite electrodes: A binder-free approach. RSC Adv..

[158] Clerici F., Fontana M., Bianco S., Serrapede M., Perrucci F., Ferrero S., Tresso E., Lamberti A. (2016). In situ MoS_2_ decoration of laser-induced graphene as flexible supercapacitor electrodes. ACS Appl. Mater. Interfaces.

[159] Mandal M., Ghosh D., Kalra S., Das C. (2014). High performance supercapacitor electrode material based on flower like MoS_2_/reduced graphene oxide nanocomposite. Int. J. Lat. Res. Sci. Technol..

[160] Huang K.-J., Wang L., Zhang J.-Z., Wang L.-L., Mo Y.-P. (2014). One-step preparation of layered molybdenum disulfide/multi-walled carbon nanotube composites for enhanced performance supercapacitor. Energy.

[161] Hu B., Qin X., Asiri A.M., Alamry K.A., Al-Youbi A.O., Sun X. (2013). Synthesis of porous tubular C/MoS_2_ nanocomposites and their application as a novel electrode material for supercapacitors with excellent cycling stability. Electrochim. Acta.

[162] Fan L.-Q., Liu G.-J., Zhang C.-Y., Wu J.-H., Wei Y.-L. (2015). Facile one-step hydrothermal preparation of molybdenum disulfide/carbon composite for use in supercapacitor. Int. J. Hydrogen Energy.

[163] Kumuthini R., Ramachandran R., Therese H., Wang F. (2017). Electrochemical properties of electrospun MoS_2_@C nanofiber as electrode material for high-performance supercapacitor application. J. Alloys Compd..

[164] Nam M.S., Patil U., Park B., Sim H.B., Jun S.C. (2016). A binder free synthesis of 1D PANI and 2D MoS_2_ nanostructured hybrid composite electrodes by the electrophoretic deposition (EPD) method for supercapacitor application. RSC Adv..

[165] Thakur A.K., Choudhary R.B., Majumder M., Gupta G., Shelke M.V. (2016). Enhanced electrochemical performance of polypyrrole coated MoS_2_ nanocomposites as electrode material for supercapacitor application. J. Electroanal. Chem..

[166] Krishnamoorthy K., Veerasubramani G.K., Pazhamalai P., Kim S.J. (2016). Designing two dimensional nanoarchitectured MoS_2_ sheets grown on Mo foil as a binder free electrode for supercapacitors. Electrochim. Acta.

[167] Wu T., Zhou X., Zhang H., Zhong X. (2010). Bi_2_S_3_ nanostructures: A new photocatalyst. Nano Res..

[168] Zhao H., Tian F., Wang R., Chen R. (2014). A review on bismuth-related nanomaterials for photocatalysis. Rev. Adv. Sci. Eng..

[169] Ma L., Zhao Q., Zhang Q., Ding M., Huang J., Liu X., Liu Y., Wu X., Xu X. (2014). Controlled assembly of Bi_2_S_3_ architectures as Schottky diode, supercapacitor electrodes and highly efficient photocatalysts. RSC Adv..

[170] Patil S.J., Lokhande C.D. (2015). Fabrication and performance evaluation of rare earth lanthanum sulfide film for supercapacitor application: Effect of air annealing. Mater. Des..

[171] Han D., Jing X., Wang J., Yang P., Song D., Liu J. (2012). Porous lanthanum doped NiO microspheres for supercapacitor application. J. Electroanal. Chem..

[172] Bagde G., Sartale S., Lokhande C. (2003). Deposition and annealing effect on lanthanum sulfide thin films by spray pyrolysis. Thin Solid Films.

[173] Patil S., Lokhande A., Lokhande C. (2016). Effect of aqueous electrolyte on pseudocapacitive behavior of chemically synthesized La_2_S_3_ electrode. Mater. Sci. Semicond. Process..

[174] Tu C.-C., Lin L.-Y., Xiao B.-C., Chen Y.-S. (2016). Highly efficient supercapacitor electrode with two-dimensional tungsten disulfide and reduced graphene oxide hybrid nanosheets. J. Power Sources.

[175] Mayorga-Martinez C.C., Ambrosi A., Eng A.Y.S., Sofer Z., Pumera M. (2015). Transition metal dichalcogenides (MoS_2_, MoSe_2_, WS_2_ and WSe_2_) exfoliation technique has strong influence upon their capacitance. Electrochem. Commun..

[176] Bissett M.A., Worrall S.D., Kinloch I.A., Dryfe R.A. (2016). Comparison of two-dimensional transition metal dichalcogenides for electrochemical supercapacitors. Electrochim. Acta.

[177] Chhowalla M., Shin H.S., Eda G., Li L.-J., Loh K.P., Zhang H. (2013). The chemistry of two-dimensional layered transition metal dichalcogenide nanosheets. Nat. Chem..

[178] Zou X., Zhang Y. (2015). Noble metal-free hydrogen evolution catalysts for water splitting. Chem. Soc. Rev..

[179] Xue M.-Z., Fu Z.-W. (2006). Lithium electrochemistry of NiSe_2_: A new kind of storage energy material. Electrochem. Commun..

[180] Wang S., Li W., Xin L., Wu M., Long Y., Huang H., Lou X. (2017). Facile synthesis of truncated cube-like NiSe_2_ single crystals for high-performance asymmetric supercapacitors. Chem. Eng. J..

[181] Arul N.S., Han J.I. (2016). Facile hydrothermal synthesis of hexapod-like two dimensional dichalcogenide NiSe_2_ for supercapacitor. Mater. Lett..

[182] Yu B., Liu W., Chen S., Wang H., Wang H., Chen G., Ren Z. (2012). Thermoelectric properties of copper selenide with ordered selenium layer and disordered copper layer. Nano Energy.

[183] Kumar P., Singh K., Srivastava O. (2010). Template free-solvothermaly synthesized copper selenide (CuSe, Cu_2−x_Se, β-Cu_2_Se and Cu_2_Se) hexagonal nanoplates from different precursors at low temperature. J. Cryst. Growth.

[184] Pazhamalai P., Krishnamoorthy K., Kim S.J. (2016). Hierarchical copper selenide nanoneedles grown on copper foil as a binder free electrode for supercapacitors. Int. J. Hydrogen Energy.

[185] Li L., Gong J., Liu C., Tian Y., Han M., Wang Q., Hong X., Ding Q., Zhu W., Bao J. (2017). Vertically oriented and interpenetrating CuSe nanosheet films with open channels for flexible all-solid-state supercapacitors. ACS Omega.

[186] Shinde S., Ghodake G., Dubal D., Patel R.V., Saratale R., Kim D.-Y., Maile N., Koli R., Dhaygude H., Fulari V. (2017). Electrochemical synthesis: Monoclinic Cu_2_Se nano-dendrites with high performance for supercapacitors. J. Taiwan Inst. Chem. Eng..

[187] Balasingam S.K., Lee J.S., Jun Y. (2016). Molybdenum diselenide/reduced graphene oxide based hybrid nanosheets for supercapacitor applications. Dalton Trans..

[188] Huang K.-J., Zhang J.-Z., Cai J.-L. (2015). Preparation of porous layered molybdenum selenide-graphene composites on Ni foam for high-performance supercapacitor and electrochemical sensing. Electrochim. Acta.

[189] Huang K.-J., Zhang J.-Z., Fan Y. (2015). Preparation of layered MoSe_2_ nanosheets on Ni-foam substrate with enhanced supercapacitor performance. Mater. Lett..

[190] Karade S.S., Sankapal B.R. (2017). Two dimensional cryptomelane like growth of MoSe_2_ over mwcnts: Symmetric all-solid-state supercapacitor. J. Electroanal. Chem..

[191] Wang Z., Sha Q., Zhang F., Pu J., Zhang W. (2013). Synthesis of polycrystalline cobalt selenide nanotubes and their catalytic and capacitive behaviors. CrystEngComm.

[192] Kong D., Wang H., Lu Z., Cui Y. (2014). CoSe_2_ nanoparticles grown on carbon fiber paper: An efficient and stable electrocatalyst for hydrogen evolution reaction. J. Am. Chem. Soc..

[193] Banerjee A., Bhatnagar S., Upadhyay K.K., Yadav P., Ogale S. (2014). Hollow Co_0.85_Se nanowire array on carbon fiber paper for high rate pseudocapacitor. ACS Appl. Mater. Interfaces.

[194] Peng H., Ma G., Sun K., Zhang Z., Li J., Zhou X., Lei Z. (2015). A novel aqueous asymmetric supercapacitor based on petal-like cobalt selenide nanosheets and nitrogen-doped porous carbon networks electrodes. J. Power Sources.

[195] Liu C.-C., Song J.-M., Zhao J.-F., Li H.-J., Qian H.-S., Niu H.-L., Mao C.-J., Zhang S.-Y., Shen Y.-H. (2012). Facile synthesis of tremelliform Co_0.85_Se nanosheets: An efficient catalyst for the decomposition of hydrazine hydrate. Appl. Catal. B Environ..

[196] Zhao X., Li X., Zhao Y., Su Z., Wang R. (2017). Facile synthesis of tremelliform Co_0.85_Se nanosheets for supercapacitor. J. Alloys Compd..

[197] Gong C., Huang M., Zhou P., Sun Z., Fan L., Lin J., Wu J. (2016). Mesoporous Co_0.85_Se nanosheets supported on Ni foam as a positive electrode material for asymmetric supercapacitor. Appl. Surf. Sci..

[198] Bhat K.S., Shenoy S., Nagaraja H., Sridharan K. (2017). Porous cobalt chalcogenide nanostructures as high performance pseudo-capacitor electrodes. Electrochim. Acta.

[199] Zhang Y., Pan A., Wang Y., Cao X., Zhou Z., Zhu T., Liang S., Cao G. (2017). Self-templated synthesis of n-doped CoSe_2_/C double-shelled dodecahedra for high-performance supercapacitors. Energy Storage Mater..

[200] Zhang C., Yin H., Han M., Dai Z., Pang H., Zheng Y., Lan Y.-Q., Bao J., Zhu J. (2014). Two-dimensional tin selenide nanostructures for flexible all-solid-state supercapacitors. ACS Nano.

[201] Guo K., Cui S., Hou H., Chen W., Mi L. (2016). Hierarchical ternary Ni–Co–Se nanowires for high-performance supercapacitor device design. Dalton Trans..

[202] Yu N., Zhu M.-Q., Chen D. (2015). Flexible all-solid-state asymmetric supercapacitors with three-dimensional CoSe_2_/carbon cloth electrodes. J. Mater. Chem. A.

[203] Patil S.J., Bulakhe R.N., Lokhande C.D. (2015). Nanoflake-modulated La_2_Se_3_ thin films prepared for an asymmetric supercapacitor device. ChemPlusChem.

[204] Xia C., Jiang Q., Zhao C., Beaujuge P.M., Alshareef H.N. (2016). Asymmetric supercapacitors with metal-like ternary selenides and porous graphene electrodes. Nano Energy.

[205] Peng H., Zhou J., Sun K., Ma G., Zhang Z., Feng E., Lei Z. (2017). High-performance asymmetric supercapacitor designed with a novel NiSe@MoSe_2_ nanosheet arrays and nitrogen-doped carbon nanosheet. ACS Sustain. Chem. Eng..

[206] Peng H., Wei C., Wang K., Meng T., Ma G., Lei Z., Gong X. (2017). Ni_0.85_Se@MoSe_2_ nanosheet arrays as the electrode for high-performance supercapacitors. ACS Appl. Mater. Interfaces.

